# Critical biblical studies via word frequency analysis: Unveiling text authorship

**DOI:** 10.1371/journal.pone.0322905

**Published:** 2025-06-03

**Authors:** Shira Faigenbaum-Golovin, Alon Kipnis, Axel Bühler, Eli Piasetzky, Thomas Römer, Israel Finkelstein

**Affiliations:** 1 Department of Mathematics and Rhodes Information Initiative, Duke University, North Carolina, United States of America; 2 School of Computer Science, Reichman University, Herzliya, Israel; 3 Department of Biblical Texts, Protestant Faculty of Theology of Paris, France; 4 School of Physics and Astronomy, Sackler Faculty of Exact Sciences, Tel Aviv University, Israel; 5 Chair of the Bible in Its Contexts, Collège de France, France; 6 School of Archaeology and Maritime Cultures, University of Haifa, Israel; Israel Antiquities Authority, ISRAEL

## Abstract

The Bible is the product of a complex process of oral and written transmissions that stretched across centuries and traditions. This implies ongoing revision of the “original” or oldest textual layers over the course of hundreds of years. Although critical scholarship recognizes this fact, debates abound regarding the reconstruction of the different layers, their date of composition and their historical backgrounds. Traditional methodologies have grappled with these challenges through textual and diachronic criticism, employing linguistic, stylistic, inner-biblical, archaeological and historical criteria. In this study, we use computer-assisted methods to address the question of authorship of biblical texts by employing statistical analysis that is particularly sensitive to deviations in word frequencies. Here, the term “word” may be generalized to “n-gram” (a sequence of words) or other countable text features. This paper consists of two parts. In the first part, we focus on differentiating between three distinct scribal corpora across numerous chapters in the Enneateuch, the first nine books of the Bible. Specifically, we examine 50 chapters labeled according to biblical exegesis considerations into three corpora: the old layer in Deuteronomy (D), texts belonging to the “Deuteronomistic History” in Joshua-to-Kings (DtrH), and the Priestly writings (P). For pragmatic reasons, we chose entire chapters, in which the number of verses potentially attributed to different authors or redactors is negligible. Without prior assumptions about author identity, our approach leverages subtle differences in word frequencies to distinguish among the three corpora and identify author-dependent linguistic properties. Our analysis indicated that the first two scribal corpora — (D, the oldest layers of Deuteronomy, and DtrH, the so-called Deuteronomistic History) — are much more closely related to each other than they are to the third, (P). This observation aligns with scholarly consensus. In addition, we attained high accuracy in attributing authorship by evaluating the similarity of each chapter to the reference corpora. In the second part of the paper, we report on our use of the three corpora as ground truth to examine other biblical texts whose authorship is disputed by biblical experts. Here, we demonstrate the potential contribution of insights achieved in the first part. Our paper sheds new light on the question of authorship of biblical texts by offering interpretable, statistically significant evidence of the existence of linguistic characteristics in the writing of biblical authors/redactors, that can be identified automatically. Our methodology thus provides a new tool to address disputed matters in biblical studies.

## 1. Introduction

The Hebrew Bible is a product of a centuries-long literary process, of texts (some transmitted orally) composed and revised by divergent authors [1–5]. One of the main questions raised in biblical studies is the identification of the authors and redactors of these texts. Such identification is necessary for historical understanding, literary analysis and theological interpretation of the biblical texts. Traditionally, scholars identify the authorship of biblical texts and their redactional layers through observations based on language, style, inner-biblical logic and geographical, archaeological and historical information. This identification helped to distinguish different groups of authors and redactors. Nevertheless, despite hundreds of years of critical scholarship, many questions have remained unanswered, and different theories exist, mainly regarding the formation of the Pentateuch and the so-called Early Prophets. The latter, together with the Book of Deuteronomy, have often been considered to constitute a “Deuteronomistic History.”

With the advent of the digital revolution, researchers recognized the importance of computerized analysis of biblical texts. Radday’s work was particularly significant; he distinguished three major authors in Chapters 1–23, 40–48 and 49–66 in the Book of Isaiah. Although this result somewhat contradicted the classical idea of a Second Isaiah in Chapters 40–55, it was compatible with the historical critical tripartition of the book. Similarly, for the Book of Genesis, Radday’s analysis (carried out with colleagues from the University of Haifa), confirmed the existence of an independent Priestly source, and invalidated the possibility of distinguishing between “Yahwist” and “Elohist” sources (see [6–8], for a much smaller corpus [9]). These were pioneering studies which were restricted by the computational capabilities of their time.

With the rise of Digital Humanities, numerous methods have been developed to address the question of authorship in modern and ancient texts [10–21] (see additional discussion in the Supporting Information). Usually, identifying authorship starts with ground-truth data, that is, homogenous corpora of *known* authorship. When a new document of *unknown* authorship is introduced, the aim is to associate it with one of the known-authorship corpora, or to conclude that it is unlikely to be represented in the data [22]. Since a single author can write texts on divergent topics using different genres [23], a major challenge in authorship studies is the identification of features characteristic of the author, and at the same time unrelated to topic or genre [10,15]. Several recent studies have applied modern techniques in machine learning and Natural Language Processing (NLP), such as Artificial Neural Networks (NN) [24–25]. Still, despite apparent progress in authorship attribution methods and their application to the Bible [13,14,18–21,26,27], many questions remain unresolved.

Distinguishing between biblical authors is more complex than it is in modern literature. This is so because almost all biblical texts are multilayered (revised or edited, and re-edited over long time periods), and hence pose a challenge for characterizing the primary composition. Therefore, supervised learning techniques are typically unsuitable as they require substantial amounts of ground-truth data. In addition, insights provided by an authorship study are arguably only useful when the tools involved are transparent and explainable. Several other issues should be noted: First, the chapter system is a late innovation and therefore cannot be taken as a basis for authorship consideration; second, chapters are short and may therefore pose a problem for robust statistical inference; third, there are challenges that are solely introduced by the Hebrew language (as opposed to the processing of English texts).

In this paper, we harness a distinctive collaboration between biblical scholars and statisticians to address the question of authorship quantitatively: First, we determine whether biblical authors can be characterized by distinct linguistic properties. We then examine whether those properties can be utilized to estimate the likelihood of attributing a given text to one of the identified authors.

The key contribution of this paper is in offering scholars a new procedure for analyzing authorship in the Bible based on lemma n-gram frequencies. We do this by employing a method that is sensitive to differences between authors, associated with potentially rare and subtle deviations in their usage patterns. This method, introduced in [28,29], has interesting optimality properties [30] and is based on the notion of Higher Criticism in statistics [31–32] (to differ from the same term used in textual research [33]). Unlike previous studies based on word n-gram frequencies [10,15,34], it has no tunable parameters and does not require mapping the data to a predefined set of manually selected attributes. This ensures that the results are interpretable and free of the researcher’s subjective influence. Furthermore, it also identifies a set of lemma n-grams which furnishes the best evidence of authorship discrimination, thus offering a valuable tool for identifying authorship similarity or discrimination. Our analysis provides attribution likelihood and identifies a list of distinguishing lemmas, enhancing the interpretability of the results.

A second contribution of this paper is the method’s sensitivity analysis – the paper performs the first trial of this method*.* For example, it was previously unclear if (and how well) the method could be applied to short texts; whether it depended on the reference corpora; and how sensitive it was to the statistical properties of words. In the “Robustness Assessment” section we discuss the test performed under these scenarios and characterize its success rate.

The first part of our project focused on the first nine books of the Bible. Based on conventional wisdom in biblical exegesis, we assembled three groups of chapters (referred to below as “corpora”) to serve as ground-truth data for the study: (a) *the oldest layers in Deuteronomy* (the so-called “Ur-Deuteronomy; hereafter D), usually dated to late monarchic times in the late 7th century BCE (e.g., [35] p. 231; [36] p. 34); (b) *the early layers in the so-called Deuteronomistic History* (DtrH – the term applied to the Books of Deuteronomy, Joshua, Judges, 1 and 2 Samuel and 1 and 2 Kings), dated to late monarchic times in the late 7th century or to the exilic period in the 6th century BCE [4]; (c) the so-called *Priestly writings* (P), commonly dated to late exilic or post-exilic times (see [37], p. 93). We assumed that each corpus represents an author or authors/redactors with distinctive cultural/ideological background. Although it cannot be assumed that the chosen chapters were free of later editorial layers (for the DtrH, see [4]), there is general agreement that an important revision of Deuteronomy had taken place when the book became part of the Deuteronomistic History or Dtr Library. However, there is also consensus that the so-called DtrH is multilayered, which made it impossible to ascribe every added passage to a specific layer. Regarding the Book of Deuteronomy, we pragmatically made a basic distinction between the original Deuteronomy, the Deuteronomistic revision and the post-Deuteronomistic revision in the context of the edition of the Pentateuch (on this see also [35]). This part of the study confirms the conventional wisdom in biblical text analysis and hence solidifies the ground-truth corpora.

In the second part of our study, we applied our methodology to additional biblical texts whose attribution is disputed. We examined their literary characteristics as compared to the three ground-truth corpora. We looked at the late Abraham material and early Jacob story in Genesis, the Ark Narrative in 1 and 2 Samuel, and several chapters in Judges, 1 and 2 Chronicles, Esther and Proverbs. Our results highlight the potential of our method to shed light on contested issues in biblical studies.

### Algorithmic apparatus

In this paper, we propose an algorithmic framework for studying biblical texts written in Hebrew, consisting of the following steps:

#### Step 1. Lemma extraction.

Transformation of the words of a given biblical text to their Hebrew lemma form, utilizing predefined labeling of the Bible.

#### Step 2. Dictionary formation.

Construction of a dictionary that consists of thousands of the most frequent lemmas or n-grams (i.e., combinations of several consecutive lemmas). Henceforth, we commonly refer to lemmas and their n-grams as features.

#### Step 3. Text descriptor construction.

Replacement of each text with a frequency table, indicating the number of occurrences of each feature within it.

#### Step 4. Document-corpus discrepancy calculation.

For a pair of texts (e.g., a document and a corpus), computation of a relative discrepancy score, called HC-discrepancy [28], based on the word frequency table of each text is performed. A low score indicates that the two texts were likely written by the same author; a high score indicates authorship discrepancy.

#### Step 5. Attribution likelihood assessment.

Given a reference corpus and a new text, proposing a model for the distribution of HC values, then testing the null hypothesis $H_{0}$, that the given text and the corpus were written by the same author. The calculated p-value (p) indicates the significance of the association of the new text to the given corpus with respect to the distribution of the HC scores of the latter. If p≤0.05, we reject $H_{0}$ and accept the competing hypothesis of two different authors. Otherwise, we remain undecided as we cannot rule out the possibility that the new text was written by the corpus’s author: The two texts may still be different in terms of authorship, but our evidence is insufficient to determine this.

#### Step 6. Authorship verification and attribution.

Given a document of unknown authorship and several candidate corpora of different authorships, calculating the p-value as described in Step 5 for each corpus. Attributing the document to the most likely author among the corpora that are not rejected with a significant 0.05. If no such corpus exists, declaring that the document cannot be associated with any of the corpora.

#### Step 7. Interpreting authorship distinction.

Analyzing the method’s decision for two different authors by identifying sets of features that drive the highest HC-discrepancy.

Steps 1–3 above are associated with pre-processing while Steps 4–7 relate to inference. The result of Steps 5–6 is a table that contains the p-values, summarizing the evidence for the verification (proximity/discrepancy) of each text to one of the corpora. The novelty of our approach is the attribution of texts pertaining to the Hebrew Bible in an interpretable manner that delivers accurate results even for relatively short texts. The power of our methodology lies in the following three stages: (a) assessing the likelihood of attributing a new text to a given corpus; (b) finding the corpus that is more likely to be written by the same scribe; (c) providing the reasoning for the text attribution (a list of features). To the best of our knowledge, there is no other published automatic analysis of biblical texts that provides an interpretable attribution on the scale and rigor reported here.

## 2. Materials and methods

### 2.1 Dataset

Each text in our dataset is accompanied by a label that associates it with one of the three corpora. For practical reasons, we worked with chapters, acknowledging that they represent a late organization of the text. We were aware that most chapters are multilayered, but for pragmatic reasons chose entire chapters, recognizing that the number of verses potentially attributed to different authors or redactors did not affect the result. Considerations for selection of ground-truth chapters were:

- They had to contain a sufficient number of words for statistical examination; for this reason, shorter passages, which contained only a few verses, although commonly considered to belong to D, DtrH or P, were not integrated into the analysis.
- They had to feature minimal stratigraphy (layers), although they were rarely uniform.
- Their classification into the three corpora had to have as wide as possible a consensus among biblical scholars.

The texts selected for the three corpora consist of the following chapters

#### Deuteronomy [D].

Deut 6; 12–13; 15–16; 18–19; 26; 28.

#### Deuteronomistic History [DtrH].

Deut 8–11; 27; Josh 1; 5; 6; 12; 23; Judg 2; 6; 2 Sam 7; 1 Kgs 8; 2 Kgs 17:1–21; 2 Kgs 22–25 (note, 2 Kgs 17 is the only passage that is not a whole chapter since; scholars agree that its second part belongs to late redactions in the Persian period);

#### Priestly texts [P].

Gen 17; Exod 6; 16; 25–31; 35–40; Lev 1–4; 8–9.

In order to compare similar genres, we opted for narration chapters. As no P chapters could be taken as pure narration, to complete our dataset we added P narration verses (to differ from chapters) from Genesis and Exodus. These were used only as reference data for the HC calculation and were not tested for author attribution (they were too short to be securely attributed). A complete list of all the verses is provided in the Supporting Information. The narration material now forms a substantial part (41%) of the P corpus (there are 289 verses of narration texts and 695 verses of other genres). The length of these texts appears in Table S1-S2 and Fig S1 there in S1 Appendix.

Since the so-called *Ur-Deuteronomium* was heavily revised, determining sufficiently long biblical texts that could be attributed to the D corpus was a challenging task. This led us to select only nine texts. Still, the total number of lemmas in this corpus was sufficient for our statistical analysis. For the widely accepted reconstruction of an original layer of Deuteronomy (D) dating back to the 7^th^ century BCE, we followed Preuss (see [36], pp. 46–61). As for DtrH, only the passages that are primarily DtrH compositions were selected (although Joshua 6 may contain an earlier kernel, and Joshua 5 may contain some post-dtr revisions cf. [4]). Texts like the royal annals or other pre-DtrH documents that were used by the Deuteronomistic authors were avoided. For the Priestly texts (P), we followed Köckert’s and Nihan’s hypotheses, which are now widely accepted in European scholarship for the end of P^g^ in Leviticus 16 [38,39], and avoided the later Priestly texts and texts attributed to the so-called Holiness-school (H). Even if there is a debate on the distinction between P^g^ (the basic Priestly layer, *Grundschrift*) and P^s^ (secondary Priestly additions), especially in Exodus 35–40 and Leviticus 1–3; 8–9, almost all scholars agree with regard to attributing these texts to the Priestly milieu. Unlike older scholarship (Wellhausen, Noth, etc.) and along with Nihan and others, we do not distinguish between the narrative and legal/ritual texts of P, because they belong to the same milieu.

### 2.2 Feature extraction

While, of late, there has been growing interest in the application of machine learning techniques to analyze the morphology of ancient texts and scripts [40–41], it remains for the medium at hand to tailor the method. Implementing accurate comparisons and characteristics is necessary. Choosing features for text comparison and authorship attribution must play a key role since it should capture the unique aspects of each text. While various features that could be used exist (e.g., [10,15,42–44]), our analysis relies on occurrences of word n-grams that fall under the so-called “bag-of-word” approach, which is also the key idea in TF-IDF, that examines the relevance of key-words to perform text classification [45–46]. One of the advantages of n-gram occurrences is its ability to apply statistical tools that are perfectly transparent; another is the relatively straightforward interpretation of the results. In this regard, our methodology provides an improvement over previous studies that handcrafted lists of words to be considered as features and imposed assumptions on the distributions of individual words [10,15,18,19,34].

As per the list given above, here is a detailed explanation of the steps in our methodological framework.

#### Step 1. Lemma extraction.

Extracting the lemmas from each document within each corpus. Here, we relied on the Open Scriptures Hebrew Bible (OSHB) project which provides lemma and morphological information [47]. We note that extracting lemmas leads to higher counts but fewer features, as there are more words than lemmas. We also note that this approach enhances the detection of deviations linked to the occurrence of a particular lemma. On the other hand, we realized that by counting lemmas we might lose a potential authorship signal, i.e., the different ways various authors use the same lemma. However, when texts are relatively short, as in our study, the benefit of improving the signal in each feature appears to overcome the loss of information. Additionally, all lemmas representing proper names and gentilic nouns were replaced with a designated code. This is based on the rationale that such lemmas are more likely to reflect the context of the text rather than its author.

Thus, once the lemmas are extracted by the OSHB, a unique number is associated with each lemma, referring to its form (e.g., lemma number 8085 refers to the word šmꜥ [to hear]). This ID is used to simplify the HC calculation. For example, the words שמענו, כשמעכם, תשמעו, וישמע, ישמעו, הנשמע (hᵃnišmaꜥ, yišmꜥû, wayyišmaꜥ, tišmꜥû, kᵉšomꜥᵃkem, šᵉmāꜥēnû) will have the same lemma (ID 8085); the underlined words are split into two distinct lemmas, reflecting their separate linguistic or semantic components. The words that correspond to lemma ID 7223 (translated as first) provide an additional example: **והראשון**, בראשנה, ראשנים, כראשנים (wahāriʾšôn, bāriʾšōnâ, kāriʾšōnim, riʾšōnim). While the bold word here results in three lemmas (ו, ה, ראשון,(, similar to the above, the two underlined words are splatted into two lemmas, each with its own ID. In the following, we use the term n-grams to refer to sequences of several consecutive lemmas. For example וישמע, אשר  צוה, אתה ו, are bi-grams. We uploaded the lemmas extracted from all the chapters to an online repository [48]; more details about the repository can be found in the readme file at the same link.

#### Step 2. Dictionary formation.

Constructing a dictionary that consists of all lemmas in the three corpora. In total, the dictionary has 1,447 unique lemmas (594 in D, 821 in DtrH, and 846 in P, with an overlay between the dictionaries).

#### Step 3. Text descriptor construction.

Counting the occurrences of each lemma in the dictionary in each text and summarizing the counts in a table (histogram). Henceforth, we refer to all entries in the table simply as “words” and to the corresponding table as a word-frequency table, with the understanding that the term “word” may have a broader context in our setting (lemma, proper names and gentilic noun codes, and lemma/code n-grams).

### 2.3 Authorship attribution via statistical analysis

#### Step 4. Document-corpus discrepancy calculation.

Evaluating an index of *discrepancy between two given texts.* We followed the method proposed by Kipnis and Donoho [28–30] that measures the resemblance of two word-frequency tables by means of calculating the Higher Criticism (HC) of many two-sample binomial tests. This approach leads to a discrepancy index, denoted as the *HC-discrepancy*, that is, an index sensitive to deviations in the occurrence frequency of a few words within a potentially vast dictionary—the identity of which was not known to us in advance [29]. The HC method, which was introduced in papers by Donoho and Jin in [31,49], does not require a-priori word selection.

**Fig. 1:**
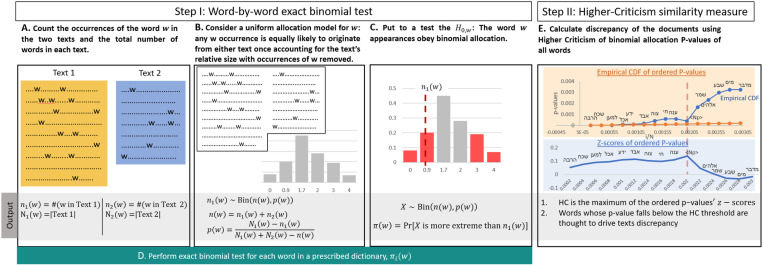
Workflow for comparing Text 1 and Text 2. Step I: Perform exact binomial testing for each word, measuring the fit of the word’s occurrences to a binomial allocation model. Step II: Conduct Higher Criticism (HC) on the per-word binomial allocation p-values and use it as an index of discrepancy between the texts. HC assesses the global significance of the p-values by comparing their z-scores to the uniform empirical process. Words associated with p-values smaller than the HC threshold are considered to provide meaningful discrimination between Text 1 and Text 2.


For the sake of completeness, we detail the HC method here; its main steps are outlined in Fig1:

For a given pair of texts $T_{1}$ and $T_{2}$, we conducted a word-by-word exact binomial test. Let N(w|T) be the number of the occurrences of the word w in text T (Fig 1, Step I, A). For each word w in the dataset a p-value is calculated by quantifying the deviation in the word’s occurrence from what is expected under a binomial allocation model:

$$H_{0,w}\;:\;N\left(w|T_{1}\right)\sim \text{Bin}\left(n_{w},q_{w}\right),\;\;\;\;\;\;\;$$ (1)

where Bin(n,p) is the binomial distribution with n trials and success probability p of each trial. Our parameters for the binomial distribution are: (a) $n_{w}$, which represents the total number of occurrences of the word in both documents, calculated as $n_{w}=N\left(w|T_{1}\right)+N\left(w|T_{2}\right)$ ; **(b)** $q_{w}$, which is the proportion of words in that document (excluding the current word) relative to the total number of words in both documents. This can be calculated as $q_{w}=\frac{\sum_{w\;\;\;\prime \;\;\;\in W,w\;\;\;\prime \;\;\;\neq w}N\left(w\;\;\;\prime \;\;\;|T_{1}\right)}{\sum_{w\;\;\;\prime \;\;\;\neq w\in W}n_{w\;\;\;\prime \;\;\;}}$. The hypothesis $H_{0,w}$ refers to the binomial allocation model of a word w across the two documents. It asserts that occurrences of w are independent, with each instance being equally likely to come from either $T_{1}$ or $T_{2}$. This is based solely on the relative size of $T_{1}$ compared to $T_{1}$ minus the occurrences of w (Fig 1, Step I, B-C).

Let ${\{\pi \left(w_{i}|T_{1},T_{2}\right\}}_{w_{i}\in W}$ be a list of the p-values as calculated from (1) for each word in the joined vocabulary, sorted in increasing order (Fig 1, Step I, D). Define Higher-Criticism by measuring the global significance of these p-values


$$HC\;\left({\left\{\pi \left(w_{i}|T_{1},T_{2}\right)\right\}}_{i=1}^{N}\right)=\underset{1\leq i\leq \gamma_{0}N}{\text{max}}\;\sqrt{N}\frac{\left(\frac{i}{N}-\pi \left(w_{i}|T_{1},T_{2}\right)\right)}{\sqrt{\frac{i}{N}\times \left(1-\frac{i}{N}\right)}}$$ (2)

where $\gamma_{0}$ is a tunable parameter. The HC score maximizes the difference between the binomial proportion $\pi \left(w_{i}|T_{1},T_{2}\right)$ to the expected value $\frac{i}{N}$ across all the words $w_{i}$ (Fig 1, Step II). The HC method appears to perform well even with a small number of reference texts that are relatively short (with the shortest text consisting of around 300 words, and the median text length of 590 words). Additionally, it can identify discriminating signals hidden in only a few (10-30) words out of possibly thousands of words while providing statistical interpretations that are well-understood.

In the paper [28], authorship study was performed on real data in the Federalist Papers and demonstrated that even such a set typically contains more author-characteristic words than topic-related ones. An additional advantage of the HC discrepancy method is that it automatically identifies a set of words that provides the best evidence for authorship discrimination via the so-called HC thresholding mechanism [31,49] as described in the right-hand side of Fig 1. As part of an overview of the PAN conference on authorship verification challenge in 2020 [50], the HC-discrepancy method was compared to other such methods and was found to attain competitive results. We adopted the HC method for this study due to its simplicity, interpretability and statistical significance properties.

#### Step 5. Attribution likelihood assessment.

Considering the likelihood that a new text can be associated with a given corpus. For the working hypothesis that the two texts were written by *different* authors, we tested the null hypothesis $H_{0}$, that the given text and the reference corpus were written by the same author. Thus, for a given document and a corpus, we considered the extended corpus, which is the union of both. Using the HC-discrepancies of the extended corpus, we estimated the distribution of the HC scores of the corpus (resulting in the mean and standard deviation of the HC values). Next, we proposed a model in which the HC discrepancies of different documents with respect to their true corpus are independent and normally distributed. We calculated a P-value p to assess the significance of the attribution of the new text to the given corpus, based on the corpus estimated distribution using a t-test. If p≤0.05, we reject the null hypothesis H_0_ and support the alternative hypothesis that the texts are from different authors; otherwise, we remain undecided.

#### Step 6. Authorship verification and attribution.

Attributing the text to the author associated with the highest t-test p-value after calculating three p-values for each text. This is the maximum likelihood decision under the probabilistic model of Step 5.

#### Step 7. Interpreting authorship distinction.

Reasoning our method’s attribution decisions. In our analysis, we calculated the HC-discrepancy values that combine the many per-word p-values into a single number, which is denoted as the HC-discrepancy between the documents. An additional by-product of the HC calculation is a set of words that have the greatest impact on the value of the HC statistic; the evidence for authorship discrimination is thought to be found there. These words permit experts to examine the outcome and understand the considerations that led to the scoring and, eventually, the attribution. Our numerical experiments were accompanied by a list of these discriminating words; their dominance in the authorship hypothesis test was evaluated in Step 4. In addition, the methodology presented in this paper is not limited to a single word’s frequencies, but also to the frequencies of n-grams (bi-gram/tri-gram).

### 2.4 Robustness assessment

We verified the robustness of our algorithmic framework using several tests. First, we tested the robustness of the method against thematic biases in the chapters. Let N be the number of unique lemmas in the reference corpus and let us create a new text by randomly sampling N unique words with repetition from the original 50 chapters while preserving the original word frequency statistics (if a word is repeated more than once we added a running index, i.e., “word_1”, “word_2”). Next, the HC-attribution method was applied to these simulated texts, and attributed to one of the three corpora. The success rate of such 100 bootstrap iterations, yielded a standard deviation of 4%. In other words, the accuracy of the HC attribution method deviates by 4% from the vanilla ground-truth attribution (84%). This indicates that the method is robust with respect to small changes in the data.

Next, we raised the question of *whether the method is dependent on the ground-truth data*. We applied k-fold cross-validation, with k = 4, i.e., by randomly splitting our ground-truth data (50 chapters) into four subgroups. This was achieved by sampling from each corpus separately, ensuring balanced sample from the reference corpora (with $\lfloor \;\;\;\;\frac{9}{4}\rceil \;+\lfloor \;\;\;\;\frac{19}{4}\rceil \;+\lfloor \;\;\;\;\frac{22}{4}\rceil \;=13$ for each group, where the ⌊ x⌉   , rounding x to the nearest integer). In each iteration, we randomly selected one group to serve as validation and used the other three groups as the reference ground-truth data (i.e., 3·13 = 39 chapters). Next, we attributed each document from the validation data to one of the existing corpora (D, DtrH or P) with respect to the 39 chapters. Executing 130 random splits resulted in an accuracy mean of 85.8% with a standard deviation of 5% across repetitions. In addition, we verified that the accuracy is robust with respect to the selected feature, i.e., single term, bigram, trigram. Indeed, the accuracy remained consistent.

Finally, we evaluated the accuracy of the HC-attribution with respect to the length of the tested texts (in terms of the number of verses in a text). Our analysis showed that even for relatively short chapters, 10 verses long, we reached 80% accuracy, which is excellent for such a small number of words. For additional details about these tests, see the Supporting Information.

## 3. Results

### 3.1 Likely or not: attributing ground-truth data

**Fig 2:**
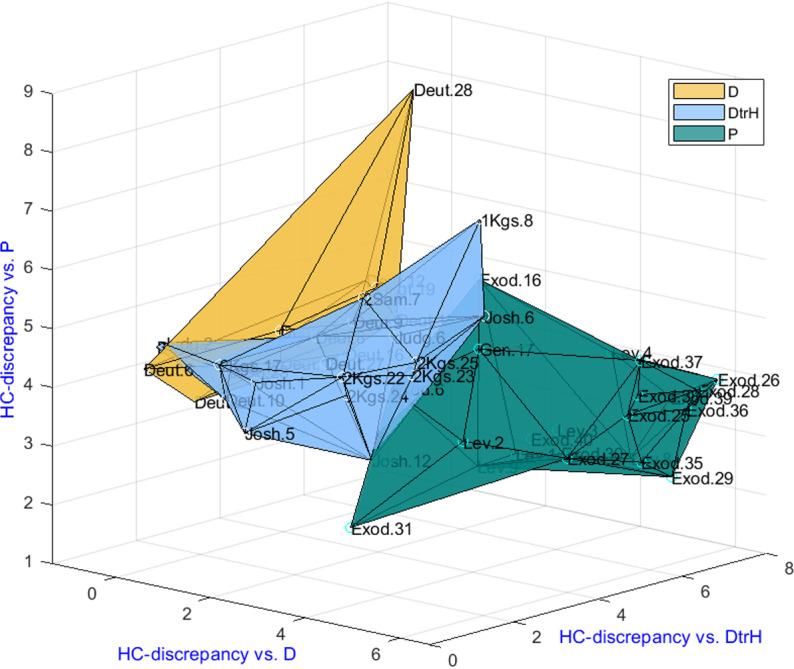
Examined biblical data displayed using the HC-discrepancy values. Each point corresponds to a chapter, indicating its HC-discrepancy with respect to each of the corpora (D, DtrH and P**)**. The labels on the nodes correspond to chapters. For the purpose of validation, only the convex hull of the chapters was colored, based on the ground-truth attribution (yellow for D, blue for DtrH and pine green for P).

To demonstrate the potential of the proposed algorithmic framework, the approach was applied to study the authorship of the selected 50 chapters in a leave-one-out manner. Each time a single chapter was removed, Steps 2–7 (see above) were performed and their HC-discrepancy was calculated (Step 4 above) with respect to the three corpora (containing the other 49 chapters). In Fig 2, we present a three-dimensional visualization of the chapters’ embedding using the HC-discrepancy method, where each axis represents one of the three reference corpora. Each point corresponds to a chapter, indicating its HC-discrepancy with respect to each of the reference corpora. Table S3 in the S1 Appendix shows a detailed list of all the HC values. The colors in the figure represent the ground-truth attributions, with the coloring serving purely as a label and having no impact on the experiments; yellow was used for D, blue for DtrH and pine green for P. Subsequently, the plots were regenerated for all three types of projection on any pair of axes. See Fig S4 in the S1 Appendix Supporting Information for a pairwise comparison of two ground-truth corpora based on their HC values. The outcome suggests an almost distinct separation between the three authors, yet at the same time indicating their proximity to each other.

Our analysis (see Fig S4 in the S1 Appendix) indicates that the examined chapters are grouped into three clear-cut clusters with a small overlap between them. While the first two authors (D and DtrH) are closely related, a distinct separation is evident between them and P, aligning with assessments of biblical scholars. This demonstrates that the three corpora have distinct linguistic properties.

**Fig 3:**
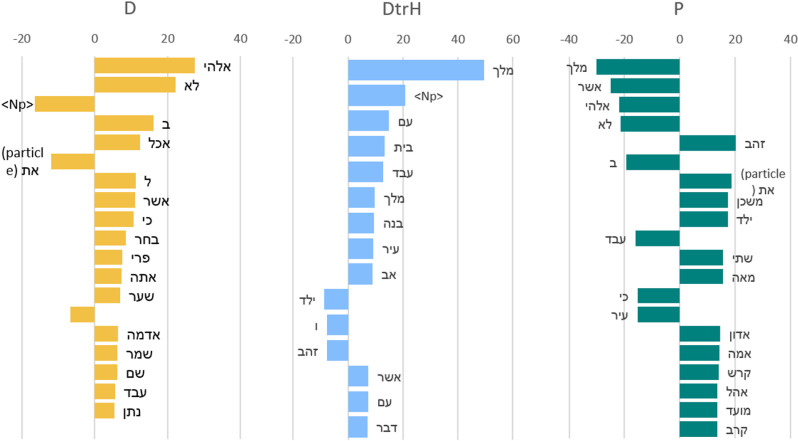
Discriminating lemmas for each corpus (D, DtrH, and P) are presented in three graphs (left to right: D, DtrH, **P)**. Each graph lists 20 lemmas (in order of importance) of a corpus vs the union of the two other corpora.

One of the main advantages of our method is that it is fully interpretable, which means that one can trace the reasoning of the HC embedding. Fig 3 presents the discriminating lemmas graphically. Each column in the graph shows a list of the 20 most discriminating lemmas for each corpus vs the union of the other two corpora as a volcano plot. The lemmas are ordered by their degree of deviation from a random allocation of occurrences in the two texts (log of the binomial allocation p-value; see the Supporting Information for an explanation). The sign of the bar indicates whether the word has high frequency in the attributed chapter (positive), or in the corpora to which it is being compared (negative). For example, discriminating lemmas for the D corpus with respect to the union of DtrH and P corpora are אלהים, לא, and < Np> (*lōʾ*, not, *ʾᵉlōhim*, God). On the other hand, the lemmas אשר, אלהים, לא, מלך (*melek*, king, *lōʾ*, not, *ʾᵉlōhim*, God, *ʾᵃšer*, which), were exceedingly frequent in D and DtrH but not in P, while the word זהב (*zāhāb*, gold) is a characterizing word for P. The HC calculations take into account both literary genre elements and milieu-specific theological expressions in determining the attribution of texts to one or the other corpora. The limited use of relative phrases (low frequency of the word *ʾᵃšer*, which) in P texts is a grammatical element that depends neither on literary genre nor on theology.

In the next step, we used the estimated three distributions of the HC scores of the ground- truth corpora to check whether a chapter was written by the same author as the given corpus (Step 5 above). In our model, we assumed that the HC discrepancies of different documents, with respect to their true corpus, were independent and normally distributed. We supported our Gaussian distribution assumption by examining the goodness-of-fit test, of whether sample data had the skewness and kurtosis that matched normal distribution using the Jarque-Bera method. We calculated the p-value of whether the distribution of the HC measures of each corpus followed the normal distribution. The results are presented in Table 1, where the examined chapters head the columns, while the examined corpora head the rows, with the intersecting cells giving the p-values p. The chapter name in the first row is colored according to the ground-truth attribution (yellow for D, blue for DtrH and pine green for P). The table provides two types of statistically significant information: 1) for a given text, we indicate whether, under the null hypothesis, it is unlikely to belong to the corresponding corpus (blue); 2) we point to the corpus to which that text can be attributed with the highest probability (orange). These findings address the main question in the text attribution by both negating potential ascriptions (to one or several reference corpora) and providing positive association answers.

**Table 1:** p-values of ground-truth chapters for the author attribution task.

	Deut 6	Deut 12	Deut 13	Deut 15	Deut 16	Deut 18	Deut 19	Deut 26	Deut 28	Deut 8	Deut 9	Deut 10	Deut 11	Deut 27
**D**	**0.97**	**0.45**	**0.64**	**0.19**	**0.38**	**0.54**	**0.47**	**0.87**	**0.23**	**0.37**	**0.16**	**0.61**	**0.90**	**0.30**
**DtrH**	**0.94**	**0.03**	**0.75**	**0.33**	**0.28**	**0.41**	**0.35**	**0.74**	**0.07**	**0.36**	**0.33**	**0.86**	**0.74**	**0.66**
**P**	**0.27**	**0.05**	**0.17**	**0.14**	**0.26**	**0.26**	**0.10**	**0.49**	**2.41E-04**	**0.10**	**0.05**	**0.41**	**0.17**	**0.23**
														
														
	**1 Kgs 8**	**2 Kgs 17**	**2 Kgs 22**	**2 Kgs 23**	**2 Kgs 24**	**2 Kgs 25**	**2 Sam 7**	**Josh 1**	**Josh 5**	**Josh 6**	**Josh 12**	**Josh 23**	**Judg 2**	**Judg 6**
**D**	**0.02**	**0.53**	**0.18**	**0.14**	**0.22**	**0.08**	**0.13**	**0.34**	**0.20**	**0.58**	**0.32**	**0.05**	**0.86**	**0.18**
**DtrH**	**0.04**	**0.91**	**0.78**	**0.41**	**0.66**	**0.49**	**0.50**	**0.99**	**0.31**	**0.98**	**0.99**	**0.10**	**0.83**	**0.34**
**P**	**0.01**	**0.12**	**0.20**	**0.25**	**0.40**	**0.27**	**0.03**	**0.24**	**0.74**	**0.30**	**0.51**	**0.12**	**0.13**	**0.17**
														
														
	**Exod 6**	**Exod 16**	**Exod 25**	**Exod 26**	**Exod 27**	**Exod 28**	**Exod 29**	**Exod 30**	**Exod 31**	**Exod 35**	**Exod 36**	**Exod 37**	**Exod 38**	**Exod 39**
**D**	**0.12**	**0.12**	**2.92E-03**	**5.07E-03**	**2.56E-03**	**7.68E-04**	**1.18E-03**	**0.02**	**0.11**	**3.26E-03**	**1.77E-03**	**1.21E-03**	**1.67E-03**	**0.01**
**DtrH**	**0.25**	**0.11**	**3.40E-04**	**2.57E-05**	**3.78E-03**	**9.03E-05**	**4.96E-05**	**2.09E-03**	**0.49**	**3.55E-04**	**3.94E-05**	**3.62E-04**	**3.59E-04**	**7.35E-05**
**P**	**0.28**	**0.02**	**0.51**	**0.30**	**0.73**	**0.39**	**0.90**	**0.74**	**0.98**	**0.80**	**0.48**	**0.13**	**0.34**	**0.36**
														
														
	**Exod 40**	**Gen 17**	**Lev 1**	**Lev 2**	**Lev 3**	**Lev 4**	**Lev 8**	**Lev 9**						
**D**	**0.04**	**0.07**	**0.01**	**0.02**	**2.90E-03**	**0.01**	**0.01**	**0.03**						
**DtrH**	**0.01**	**0.11**	**0.01**	**0.07**	**4.82E-03**	**1.20E-03**	**2.05E-04**	**0.02**						
**P**	**0.64**	**0.22**	**0.74**	**0.77**	**0.58**	**0.19**	**0.83**	**0.81**						

The table provides two types of information: (a) if p ≤ 0.05, we reject $H_{0}$, indicating that the given text is unlikely to belong to the corresponding corpus under the null hypothesis (blue). (b) each chapter is attributed to one of the corpora (D, DtrH, P) based on the maximal p-value of the three corpora (solid orange).

In the following, the calculated p-values were used in two ways: in Analysis 1, to determine the likelihood that the text can be attributed to one of the reference corpora, and in Analysis 2, identify which specific corpus it belongs to.

#### Analysis 1.

We pose the null hypothesis$\;H_{0}$, *that the given text and the tested corpus were written by the same author*. if p≤0.05, we reject $H_{0}$, and conclude that the given text and the reference corpus are unlikely to have been written by the same person (the p-value is shaded in blue in Table 1). As can be seen, the True Positive (TP) attribution rates are 0.68, 0.71, and 0.98 for the D, DtrH, and P corpora, respectively. This supports the assumption that the HC scores are normally distributed, as their spread aligns with the quantiles of the normal distribution.

For a given chapter, a False Negative (FN) occurs when the corresponding null hypothesis $H_{0}$ is rejected with respect to a particular corpus, although the chapter is attributed to that corpus by biblical experts. In our case, the only two FNs stand for the negation of the attribution of 1 Kings 8 to DtrH with p-value of 0.04, and the incorrect rejection of the attribution of Exodus 16 (that should be attributed to P) with p-value of 0.02. The latter FN rate merely agrees with the expected FN rate of 5% in our model. The low FN rate demonstrates the power of our methodology and expedites the possibility of applying it to additional biblical texts, as we will describe in the second part of this paper (below). Note that by ruling out the attribution of 1 Kings 8 to all three reference corpora (with p-values 0.02, 0.04, 0.01), 49 chapters remain for the attribution task.

#### Analysis 2.

Here, we turned to the attribution task itself (Step 6 above). For each chapter, three values were presented (in the corresponding column), each indicating the likelihood of attribution to the corresponding corpus. Subsequently, for each text we looked for the corpus that is most likely to have the same authorship, i.e., the corpus with the highest probability. For each chapter, we highlighted in orange the p-value with the highest attribution likelihood. As can be seen, in 84% of the cases, the automatic attribution coincided with biblical scholarship assessments (41 out of the 49 chapters). Since the error rate is also influenced by text length (see Fig S8 in the S1 Appendix), this can explain some of the misclassification. Analyzing the true positive accuracy for each corpus individually results in 78%, 72%, and 95% for D, DtrH and P respectively (with 7 out of 9, 13 out of 18, and 21 out of the 22 chapters). For detailed confusion matric, see Tables S4-S6 in the S1 Appendix. Not surprisingly, this indicates that it is easier to distinguish P texts from D and DtrH texts than D from DtrH. This affirms our conclusions above regarding the word usage patterns in the P corpus. Note that 5 out of the 8 (62%) misclassifications occurred between D and DtrH, which also seems logical, since the scribes of the oldest layers of Deuteronomy and the DtrH probably belonged to the same “school.” It is important to recognize that some misclassifications can arise from small differences in likelihood. For example, Joshua 23 was misclassified as P and not DtrH with a likelihood of 0.12 vs 0.10 respectively, and Judges 2 was misclassified as D instead of DtrH with a likelihood of 0.86 vs 0.83.

To better understand the reasoning for success in the attributions, we need to zoom-in on the words in each chapter that played a role in the decision. Specifically, which words indicate that Exodus 25–31 and 35–39 and corpus D (or DtrH) were not written by the same author. For example, the most important distinguishing words for Exodus 25 from D are (left to write by importance) זהב, ,קנה ,אמה טבעת, ארון (*zāhāb*, gold; *qānê*, reed; *ʾammâ*, cubit; *ṭabbaʿat*, ring; *ʾᵃrôn*, ark) while for Exodus 26 the distinguishing words from D are יריעה, קרש, משכן, ה,אחד (*yᵉrîʿâ*, curtain, *qereš*, board, *miškān*, tabernacle, *ha*, the); and from DtrH are קרש, יריעה, משכן, אדון, <Np> (*qereš*, board; *yᵉrîʿâ*, curtain; *miškān*, tabernacle, *ʾādôn*, lord). Full interpretability details are available online in the word tables in the link]48] (along with the description in the readme file). The words that distinguish P from D and DtrH in Exodus 25–31 and 35–39 are all related to the Tent of Meeting: Objects of worship such as the ark, parts of the tent such as curtains, boards, rings, materials such as gold, or elements of measurement such as cubits. All these terms appear several times in the above-mentioned chapters and other parts of the P texts which are particularly concerned with worship, clergy and the associated rites.

Herein we briefly discuss the misclassification of eight of the 49 chapters. These also appear in Fig 2 in the overlap between the corresponding classes (and in the plots of the projections in two dimension in Fig S4 in the S1 Appendix). The eight chapters for which the algorithm and the opinion of biblical scholars do not match appear in Table 2, along with the discriminating words that have the most significant effect on the value of the HC statistic; these words can enhance understanding of the reasoning for authorship rejection. In addition, see the Ground-truth chapter attribution section in the Supporting Information for a detailed discussion of these eight chapters.

**Table 2:** Summary of the discriminating words of the eight chapters for which the algorithm and the opinion of biblical scholars do not match.

	Chapter	Biblical experts’ association	HC Algorithm attribution	A set of (at most ten) “discriminating words” which have the most significant effect on the value of the HC statistic. The words are ordered from left to right by their effect level. (-) indicates negative affect.
1	Deut 13	D	DtrH	**versus D**: או (ʾô, or); הלך (hlk, to go); נדח (ndḥ, to wield); היא (hîʾ, she); ידע (ydꜥ, to know); **versus DtrH**: או (ʾô, or); קרב (qereb, midst); (-)<Np > ; נדח (ndḥ, to wield); לא (lōʾ, not);אלהים (ᵉlōhim, God); עבד (ꜥbd, to work).
2	Deut 15	D	DtrH	**versus D**: שנה (šānâ, year); אח (ʾāḥ, brother); עם (ꜥim, with); פתח (ptḥ, to open); כי (kî, that, when); בכר (bᵉkōr, first-born); שלח (šlḥ, to send); ברך (brk, to bless); **versus DtrH**: אח (ʾāḥ, brother); כי (kî, that, when); ברך (brk, to bless); לא (lōʾ, not); פתח (ptḥ, to open); בכר (bᵉkōr, first-born); (-)<Np > ; שנה (šānâ, year); בְּ (b, in); רעע (rꜥꜥ, to be bad).
3	Deut 8	DtrH	D	**versus D**: ידע (ydꜥ, to know); למען (lᵉmaꜥan, for the sake of); שכח (škḥ, to forget); מדבר (midbār, wilderness); רבה (rbh, to become many); ענה (ꜥnh, to answer); **versus DtrH**: רבה (rbh, to become many); שכח (škḥ, to forget); למען (lᵉmaꜥan, for the sake of); אכל (ʾkl, to eat); ידע (ydꜥ, to know); אבד (ʾbd, to perish); צוה (ṣwh, to summon); חיה (ḥyh, to live); ענה (ꜥnh, to answer); (-)<Np>
4	Deut 11	DtrH	D	**versus D**: את (ʾēt, < direct object>); ירש (yrš, to take possession of); יום (yôm, day); **versus DtrH**: ירש (yrš, to take possession of); (-)<Np > ; ברכה (bᵉrākâ, blessing); אנכי (ʾānōkî, I); צוה (ṣwh, to summon); ארץ (ʾereṣ, earth); מטר (māṭār, rain); יום (yôm, day); ירש (yrš, to take possession of); אנכי (ʾānōkî, I).
5	Judg 2	DtrH	D	**versus D**: שפט (špṭ, to judge); גם (gam, also); אב (ʾāb, father); אחר (ʾaḥar, after, behind); ברית (bᵉrît, covenant); עזב (ꜥzb, to leave); דור (dôr, generation); מלאך (malʾak, messenger); שסה (šsh, to plunder); **versus DtrH**: שפט (špṭ, to judge); אחר (ʾaḥēr, another); עבד (ꜥbd, to work); גם (gam, also); לא (lōʾ, not); דור (dôr, generation); עזב (ꜥzb, to leave); יד (yod, hand); שפט (špṭ, to judge); לא (lōʾ, not).
6	Exod 16	P	D	**versus D**: < Np > ; לחם (leḥem, bread); אֶל (ʾel, towards); מדבר (midbār, wilderness); אמר (ʾmr, to say); בקר (bōqer, morning); עֵדָה (ꜥēdâ, congregation); מן (mān, manna); יום (yôm, day); שַׁבָּת (šabbāt, Sabbath); **versus P**: יום (yôm, day); מן (mān, manna); לחם (leḥem, bread); מדבר (midbār, wilderness); עד (ꜥad, until); בֹּקֶר (bōqer, morning); נוח (nwḥ, to settle); ילד (yld, to give birth); אמר (ʾmr, to say); אכל (ʾkl, to eat).
7	Josh 5	DtrH	P	**versus DtrH**: מול (mwl, to circumcise); חרב (ḥereb, sword); מחר (māḥār, the morrow); תמם (tmm, to be complete); מדבר (midbār, wilderness); **versus P**: מול (mwl, to circumcise); חרב (ḥereb, sword); דרך (derek, way); בְּ (b, in); שבע (šbꜥ, to swear); שר (śar, leader); מלחמה (milḥāmâ, war); פסח (pesaḥ, passover); צור (ṣûr, rock); מחר (māḥār, the morrow).
8	Josh 23	DtrH	P	**versus DtrH**: אלה (ʾēllê, these); גוי (gôy, people); אלהים (ᵉlōhim, God); ל (l, to, for); טוב (ṭôb, good); דבק (dbq, to cling); **versus P**: אלהים (ᵉlōhim, God); גוי (gôy, people); ירש (yrš, to take possession of); טוב (ṭôb, good); אלה (ʾēllê, these); דבר (dābār, word); ל (l, to, for); שאר (šʾr, to remain); נפל (npl, to fall); אבד (ʾbd, to perish).

Following are a few comments regarding the words in Table 2: (a) A word appearing in only one text can still serve as a key indicator for author differentiation. (b) A “-” sign before a word indicates differentiation that occurred due to the reference corpus, while (omitted) positive signs indicate differentiation originating from the text being attributed. (c) We list all the indicative words that could have led to the rejection of the same-author hypothesis; however, sometimes our evidence is not sufficient to conclusively determine two distinct authors (for p>0.50).

Distinguishing between D and DtrH texts is difficult for both scholars and the algorithm, since the two corpora are closely related. Indeed, DtrH borrows certain expressions from D. This can explain the misclassification of Deuteronomy 8, 11, 13, 15, and Judges 2.

Misattributions between DtrH and P can be explained by the use of specific terms. For example, the presence of the root מול (mwl, to circumcise) in Joshua 5 was probably associated with Abrahamic circumcision in Genesis 17 (P). In Joshua 23, the repeated use of words like טוב (ṭôb, good) or אלהים (ᵉlōhim, God) appear in the P origin stories, which exclusively use this divine appellation, and Genesis 1, which repeatedly declares the creation to be “good.” Less significantly, the word אלה (ʾēllê, these) is used to open lists, and P texts also often use this term to open a narrative or a list (12 occurrences in the selected P chapters).

### 3.2 Who wrote it? Shedding light on the attribution of other biblical texts

Our primary analysis indicates a significant difference in word usage among the three ground-truth corpora and demonstrates high accuracy levels in the classification task, thus, paving the way to test the attribution of biblical texts that are disputed among scholars. In this section, we apply our automatic algorithmic framework to test nine groups of chapters vis à vis the three ground-truth corpora. Here too we selected texts that contain enough statistical information, i.e., they are long enough, with minimal evidence for editorial phases.

The selected texts are:

1. **Deuteronomy 4**: often considered to be one of the latest texts in Deuteronomy, integrating priestly language (see [35], p. 532–38).
2. **Leviticus 26:** this is the original conclusion of the Holiness Code, and is considered by many biblical scholars to be a post-Dtr and post-P chapter (see [38], p. 535–45).
3. **The Ark narrative within the DtrH:** The question here is whether the two parts of this narrative originated from the same hand [50]: (a) **Ark 1:** 1 Samuel 4–6 (this part of the narrative ends in 1 Samuel 7:1 (being short, this verse was not included in our dataset); (b) **Ark 2:** 2 Samuel 6.
4. **Chronicles:** 1 Chronicles 12:8–15, 23–40; 22:1–29:21; 2 Chronicles: 19:1–20:30; 29:3–31:21; 33:11–17; 34:3–7; 36:22–23. We have selected texts without a parallel in Samuel-Kings, which are probably late compositions (4^th^ century BCE or later, see [52]) and should therefore be distinguished from DtrH texts (see [53], p. 23–28 or [54]).
5. **Late material in the Abraham narrative:** Genesis 14–15; 20; 22; 24. Given that the different narratives of the patriarchs in Genesis were written by several redactional milieux, we wish to examine whether we can distinguish between the narratives of similar type with the same characters but different redactors. The late Abraham material seems to date to post-exilic times [55,56].
6. **The Gibeah story:** Judges 19–21. This text is considered one of the latest in the Book of Judges [57–58]. Some scholars, however, associate it with a Deuteronomistic milieu [59].
7. **The early Jacob story:** Genesis 25:7, 24–26; 28:10–22; 29:15–30; 30:25–42; 31:1–22; 46–47. The story of Jacob in Genesis is considered by most scholars to contain an early (8^th^ century BCE) storyline from the kingdom of Israel [60]. If so, this story should be distinguished from other patriarchal narrative texts.
8. **Esther:** The Book of Esther is a late narrative text whose main plot is written by a single milieu, probably during the Hellenistic period [61]. The received text has undergone modifications that can be observed by comparing the Masoretic Text, the Septuagint and the Alpha Text. Our analysis focusses on the Masoretic Text.
9. **Proverbs:** Proverbs 10–31. These chapters belong to wisdom literature, which is a literary genre different from the other selected texts.

After extracting the lemmas and building a dictionary, we calculated the HC-discrepancy between each of the above-listed texts and our three ground-truth corpora, and accordingly, the association likelihoods. Table 3 summarizes the resulting p-values. Here, too, the intersecting cells providing the p-value. When p≤0.05, i.e., the tested text is unlikely to be affiliated with the given corpus (at a significance level of 0.05 under the null hypothesis).

**Table 3:** Likelihood values of the authorship attribution of the additional texts.

	Deut 4	Lev 26	Ark 1	Ark 2	Chr	Late Abraham	Gibeah	Early Jacob	Esther	Prov
Length	1290	1004	1512	569	2081	3420	2605	1570	3066	7063
D	0.29	**0.011**	**0.038**	0.57	**6.400E-04**	**0.036**	**0.019**	**0.13**	0.05	**4.524E-06**
DtrH	**0.61**	**0.020**	**0.044**	**0.84**	**5.197E-06**	**0.010**	**0.006**	0.10	**0.06**	**3.578E-07**
P	**0.007**	**0.024**	**0.018**	0.46	**4.957E-08**	**2.084E-05**	**5.183E-05**	**1.664E-03**	**1.443E-03**	**1.961E-11**

A p ≤ 0.05, blue text, indicates that the text is not likely to be attributed to the ground-truth corpus under the null hypothesis. The maximal attribution probability for each text is given in orange.

**Based on the findings in Table 3**, **and our analysis in rejecting the null hypothesis, we see that:** (A) *Deuteronomy 4* and the early *Jacob story* are unlikely to be associated with *P*. This corresponds to the conventional position in biblical studies. The early Jacob story is far earlier than P and also differs in style. Regarding Deuteronomy 4, the integration of priestly motives or the intertextuality created by the similarity of expressions with P texts did not mislead the HC calculations which did not find these features relevant. The text of Deuteronomy 4 seems to be composed mainly of Deuteronomistic elements, hence its greater proximity with the DtrH and D texts. (B) *Esther* cannot be associated with P. (C) *Leviticus 26, the first part of the Ark narrative, the “Sondergut” of Chronicles, the late Abraham material*, the *Gibeah story* and *Proverbs 10–31* are unlikely to be associated *with either of the three ground-truth corpora*. Here, too, the results adhere to the traditional options in biblical scholarship (for example, Leviticus 26, for more details see [62–64]).

On the positive side, we observe the following possible attributions:

(A) *Deuteronomy 4* is more likely to be associated with DtrH or D.
(B) Although we were not able to negate the closeness between *Esther* and DtrH or D, the probability of such an association is very low (6% and 5%). Therefore, Esther is unlikely to be associated with either of the three corpora.
(C) For the *Early Jacob* material, we were not able to negate an association with D; this attribution has a 13% probability.
(D) Although *Ark 1* and *Ark 2* deal with the same theme and are sometimes considered to constitute one narrative [65–66] (for a history of research [67]), *Ark 1* cannot be associated with either of the three corpora, while *Ark 2* is close to DtrH. This adheres to the interpretation of *Ark 1* as an early, pre-DtrH northern text, and *Ark 2* as part of the DtrH composition [51].

The results for the texts added and compared to the three ground-truth corpora are summarized in Table S9 in the S1 Appendix. See there also the two-dimensional plots of the additional materials overlaid on the ground-truth corpora (Fig S5 in S1 Appendix), as well as the list of the discriminating words (Fig S7 in S1 Appendix).

## 4. Discussion and conclusions

Biblical materials pose specific difficulties for computational analysis, first and foremost due to their multilayered nature. This paper demonstrates that a computer-assisted method can detect patterns and offer new insights into authorship studies of biblical materials, and also foster notions achieved in the manual work of biblical scholars. The novelty of our approach is the attribution of texts in an automatic and interpretable method that delivers accurate results even for relatively short texts. This computational framework can help resolve the authorship challenge by assessing the likelihood of attributing a new text to a given corpus. Additionally, it offers researchers the possibility of deciding whether and on what grounds such a text can be attributed to a defined corpus, and of providing the reasoning for such an attribution. To the best of our knowledge, there is no published analysis of biblical texts that provides an interpretable attribution on the scale performed in this paper.

We analyzed the authorship of 50 chapters attributed by biblical experts to three ground-truth corpora (D, DtrH, and P). Our research sheds light on the authorship attribution of these chapters, as well as on additional materials, disputed in biblical scholarship.

First, we demonstrated that the three corpora can be differentiated based on word frequency usage. Using the HC-discrepancy method to embed the chosen biblical texts into a three-dimensional space, we identified three almost distinct clusters, corresponding to authors of corpora D, DtrH and P. Thus, we showed that each corpus has a unique linguistic fingerprint. Notably, we also observed that the D and DtrH chapters are closely related, while the P chapters are rather distinct.

Second, after modeling the distributions of the three ground-truth corpora, we addressed the question of whether a given text was written by the author of the tested corpus. The result shows only a 4% false rejection of the ground-truth author. This indicates that our constructed model is a good tool to describe the ground-truth corpora.

Third, we utilized the models to assess the likelihood that a given text belongs to one of the reference corpora. Using the highest likelihood value for the attribution task resulted in an 84% success rate. Specifically, the success rates for attributions to D, DtrH and P were 78%, 72%, and 95% respectively. These results were also confirmed through examination of multiple n-grams (i.e., bigrams, trigrams); see Table S10 in the S1 Appendix.

Fourth, these statistically significant results pave the way for applying the method to other biblical texts with disputed classification. In the case of the Ark Narrative, we showed distance between its two parts – Ark 1 and Ark 2 – and shed light on the debated issue of the original ending of this narrative. We also managed to confirm that Leviticus 26 shows affinities with the Priestly texts and Deuteronomy 28 (belonging to D), from which the redactors borrowed numerous expressions. The use of lexemes in the HC calculations could have been the misleading factor in associating this chapter with either P or D. Nevertheless, the result shows that taken as a whole, it is different from both corpora, as argued by biblical scholars.

Our results are accompanied by discriminating lemmas that describe the attribution, explain why it occurred, or why it was rejected. The interpretability of our method, through automatic identification of word usage patterns, is the cornerstone of our collaboration with biblical scholars, who can reveal the rationale behind the algorithm.

Three factors may affect our results, although not substantially alter them: a) the length of the attributed text; b) the corpus’s size; and c) the similarity between corpora. Regarding the first factor, the number of verses and words in chapters or passages, naturally influences the attribution task. Our analysis revealed that for texts with fewer than 10 verses, the average likelihood of correct attribution is very low, while texts with 10–30 verses had ca. 80% chance of correct attribution. In our analysis, the shortest chapter was Joshua 5 with 15 verses, while the longest was Deuteronomy 28 with 69 verses. The median chapter length was 28 verses, which, on average, resulted in an 84% accuracy rate (see Fig S8 in the S1 Appendix for a detailed analysis). This fact underscores the remarkable nature of the results presented in this paper. Indeed, we observed that the shortest chapters are more prone to incorrect attribution (e.g., Deut 13, Deut 15 - with lengths of 22–23 verses, Josh 5 and Josh 23 - with 15–16 verses, and Judg 2 with 23 verses, as shown in Table 1).

The second factor to consider is the size of the reference corpus which obviously influences the accuracy. The reference corpora differ in the amount of available data for the machine to learn the words pattern (P with 22 long chapters with median number of 35 verses, vs 8 mostly very short texts with 22 verses in D with median length of 22 verses, and 20 chapters in DtrH with 25 verses). It is reasonable to assume that the richness of the P corpus provides a more robust characterization of the author(s).

The third factor to consider is that corpora D and DtrH were relatively close in the three-dimensional space (Fig 2), making classification between them more challenging. For instance, 4 out of the 28 chapters (14%) in these two corpora were misclassified.

In this paper we demonstrated that investigating biblical texts through word/bi-gram/tri-gram frequency statistical analysis is a promising research track. This research paves the way for scholars to address additional questions in biblical studies in statistical methods, thus improving our understanding of the formation of the Hebrew Bible.

## Supporting information

S1 Appendix Methodological details on attribution method and results. (DOCX)
