# Supplementary material for: Critical biblical studies via word frequency analysis: Unveiling text authorship
Source: PLoS One. 2025 Jun 3;20(6):e0322905. doi: 10.1371/journal.pone.0322905 (PMC12132927; doi:10.1371/journal.pone.0322905)
Supplement: S1 Appendix — (DOCX) [file pone.0322905.s001.docx]

**Supplementary Information for**

**Critical biblical studies via artificial intelligence: unveiling text authorship**

Shira Faigenbaum-Golovin*, Alon Kipnis, Axel Bühler, Eli Piasetzky, Thomas Römer, Israel Finkelstein

*Corresponding author. Email: shira.golovin@math.duke.edu

**This PDF file includes:**

Supplementary Text

Figs. S1 to S8

Tables S1 to S10

# Introduction

Many methods for authorship attribution have been proposed in the past (to mention just a few [6-17], as well as [53-59]). Although most of the methods are optimized for other languages and especially for English, some of them can be carried out in different languages. However, usually, the methods require a sufficient amount of ground-truth training data (for pattern analysis or possibly by teaching Neural Networks to differentiate between writers), which is rarely available in the disputed field of biblical exegesis. In addition, often it is assumed that the texts are of a certain length. In addition, almost all of the methods are not self-explainable, in the sense that it is hard to interpret the considerations behind the attribution.

In contrast, biblical texts pose many challenges to the task of author attribution. Over the years several methods were developed to deal with the Hebrew language and with the biblical text in particular [14-15]. Aside from having been written in Hebrew, the texts are relatively short, and they are multilayered (i.e., they were edited and re-edited over the years). In addition, they are not accompanied by secure label attribution to a known writer. Therefore, in this paper, we decided to adopt the HC method to address all these challenges and also provide with interpretability. A special case of open-set attribution is authorship verification where there is only one candidate author [23]. Among author identification tasks, authorship attribution plays a key role since any given case can be decomposed into a series of authorship verification instances (as will be further discussed below).

The main goal of the current research is to study authorship attribution of biblical texts. Herein, we build on top of the observations in [18] and propose an algorithmic framework that is able to address the following three issues (a) Assessing the likelihood of attributing a new text to a given corpus. (b) Finding the corpus for a new text that is more likely to be written by the same scribe. (c) Providing the reasoning for the text attribution (a list of words) all on short texts from the Hebrew bible. The roots of the method used in this paper start with the papers by Donoho-Jin [35, 26], and continue with the enhancements introduced by Kipnis [18-19, 34]. The theoretical analysis of the statistical method performed in these works, as well as the robustness of statistical attribution performed herein, provides the grounds for the HC discrepancy performance. The current method is able to deal with a small number of reference texts, that are relatively short (with the shortest text consisting of around 300 words, and the median text length is 590). In addition, one of the main advantages of the proposed method is that it can identify discriminating signals hidden in only a few 10-30 words out of possibly thousands of words while providing statistical interpretations that are well-understood. To the best of our knowledge, there is no other publication in the literature on authorship analysis that shares these properties.

The input for our system is a list of biblical chapters and their verses that require attribution. Our algorithm involves preparatory stages, accompanied by a step that measures the relative similarity of two given texts. All the stages are fully automatic, except that the first step relies on lemmas and morphology data provided by the *Open Scriptures Hebrew Bible project* [33].

**The basic steps of the algorithm are as follows**:

**Step 1. Lemma extraction.** Convert the words in the examined texts into their Hebrew lemma form, based on the parsing provided by the *Open Scriptures Hebrew Bible project* [33]. Word prefixes such as (ל, ב, ה, ו) (*w*, and, *h*, the, *b*, in, *l*, to) are separated from the main lemma and counted separately. With this process, the person form information of a word is lost. For example, both words כליו (*kēlāyw*, his articles) and כליהם (*kᵉlêhem*, their articles) are represented by the same lemma code and hence addressed in the same manner in our approach.

**Step 2. Dictionary formation.** Create a dictionary that consists of the 3,000 most frequent lemmas by each of the D, DtrH, and P corpora, using the extracted lemmas. In the robustness examination section lemmas and n-grams will also be considered, i.e., combinations of several consecutive lemmas. Henceforth, we refer to either individual lemmas or lemmas n-grams collectively as “features.”

**Step 3. Text descriptor construction.** Create a histogram of the lemmas or n-gram occurrences (a list indicating the number of occurrences of the features in it) for each text.

**Step 4. Document-corpus discrepancy calculation.** For a document pair, compute a relative discrepancy score, called HC*-*discrepancy [18], based on the extracted features. Low HC-discrepancy values indicate authorship similarity, while high values indicate different authorship.

**Step 5. Attribution likelihood assessment.** Estimate the attribution likelihood based on the probabilistic model, by calculating the association significance.

**Step 6. Authorship verification and attribution.** We associate the text to the corpus that has it most likely to be the author under the probabilistic model we derive in Step 5. In our settings, under the model assumptions, the most likely author also has the largest p-value.

**Step 7. Interpreting authorship distinction.** Identify a set of features that provides the highest HC-discrepancy between document-corpus pairs or corpus-corpus pairs.

The novelty of this study is the attribution of Biblical texts in a transparent manner. Our framework consists of several pre-processing Steps 1-3, and a novel approach that adopts the HC-discrepancy measure to 1) Attain the likelihood of attributing a new text to a given corpus; 2) find the corpus for a new text that is more likely to be written by the same scribe; 3) provide the reasoning for the text attribution (a list of words).

An in-depth description of each of these stages is presented in the next sections. This will be followed by a description of the application of our algorithm to biblical texts. We verify the validity of our approach by applying the algorithm in various configurations and scenarios. All of the following steps are executed on the lemma or n-gram level.

# Methods

There is a growing consensus that statistical methods utilizing word-frequencies analysis can address authorship challenges [49-50]. In their study Mosteller and Wallace [32] demonstrated in their study that different authors use certain words with various frequencies, leaving the identification of these distinguishing words as an unresolved challenge in authorship studies [67-68]. The underlying idea is that authors have word-frequency fingerprints that characterize them. Therefore, the authorship of a new document can be determined based on its similarity to the word frequency fingerprints of a known author. Noteworthy, that this is also the key idea in the method TF-IDF [69-70].

The first three steps of our algorithm consist of extracting the lemmas, creating a dictionary containing the lemmas, and creating for each text the frequency table (a histogram) of its lemmas.

### **Step 1: Lemmatization**

Words tend to change when they are used in different grammatical forms. The prefix or ending of the word is replaced by a grammatical ending and this leads to a new form of the initial word. The process of removing the suffix and prefix, and identifying the normalized word form, is called *Lemmatization*. The stemming (or lemmatization) reduces different forms of a word like its noun, adjective, verb, adverb, etc. to its root form. For instance, the suffixes or prefixes of words הארץ, בארץ, ארצות *haarets, baarets, aratsot* (*hāʾāreṣ, bāʾāreṣ, ʾᵃrāṣôt*) (or in English calculating, calculates, calculated) would change to get the normalized form ארץ *arets* (*ʾereṣ*) (calculate).

In natural languages, many derived words from the same morphological class exist for grammatical and linguistic reasons. In particular, at times the count of derived words is very high due to the structure of the language. From this point of view, the lemmatization process is crucial to take the uninflected form of words for applying information retrieval (IR) or any other text processing method. it is the ‘fundamental annotation step’ that allows related word forms, often forms with extensive morphological variation, to be grouped under a single identifier.

The chapters in the Bible are very short, therefore constructing a frequency histogram will result in a histogram with only low frequencies, in the sense that most of the words will appear only once or twice. Such a scenario can challenge any method that performs word frequency comparison. To overcome the low word frequency and align better with interpretations of the HC– discrepancy we used lemmatization. In this way, the lemma frequency histogram is rich and consists of all the available word forms, and provides more robust results as opposed to using the words. Lemmatization cut down additional information on the feature layer, that could have enhanced the authorship attribution task [60]. Naturally, the grammatical information could improve the text comparison. However, for the given short texts that are examined in this case this would not result in a statistically significant comparison, since there would be only a few common grounds for it. Based on the analysis performed in the paper [34] it follows that HC-discrepancies are usually less affected by topics compared to authorship. This is also supported by the robustness tests we describe below.

Creating an automatic lemmatization is a challenging task. Usually, the developed algorithms are tailored for a specific language, with its specific suffixes and prefixes. Naturally, over the years, the English language gained a lot of attention [61-63]. A separate approach needed to be developed for Hebrew texts. In [64] the authors claim that for many morphologically-rich languages (MRLs), existing pipelines show sub-optimal performance. In recent years lemmatization algorithms were developed for Hebrew, e.g., Stanford’s CoreNLP [65], or the web application created by the ONLP lab from the Open University of Israel [64], as well as analytical tools for Hebrew texts introduced by Dicta [66]. Unfortunately, from our experience, the automatic procedures produce an unsatisfactory result for our purposes.

In our case, since our research dealt with predefined texts, another solution that utilizes a lemmatized version of the Hebrew Bible was selected. The *Open Scriptures Hebrew Bible project* offered exactly the information that is required [33]). The project contains lemma and morphology data in a very convenient XML schema form (see https://github.com/openscriptures/morphhb). Each word is labeled with a unique number, referring to its lemma form (e.g. lemma number 1121 refers to the word *bani*). For the chapters and verses, our wish to compare the first step was to load both its lemmas and the morphological information for further filtering (as will be explained later). A list of all the morphological information can be found at

https://hb.openscriptures.org/parsing/HebrewMorphologyCodes.html.

### **Step 2: Dictionary Formation**

Following the lemmatization process, the morphological information is used to replace all lemmas representing proper names by a designated code (marked as <Np> in the discriminating lemma list) in order to have a joint frequency for all the proper names within a given text regardless of what these names are. We repeated this replacement procedure for lemmas representing gentilic nouns (marked as <Ng>). The rationale for these replacements: though these types of lemmas are likely to be strongly associated with the text’s context, the frequency of appearance of all proper names or gentilic nouns may still be author-typical. Henceforth, we use the term “words”, with the understanding that the term “word” may have a broader context in our setting (e.g., n-grams, proper names, and gentilic noun codes).

Once the texts are transformed into a list of words, we constructed a dictionary that consists of the most frequent words. Specifically, the frequency of all the words was calculated and a dictionary that contained these lemmas was created.

### **Step 3: Feature Extraction**

The cornerstone feature for text similarity used in our method is the frequency of the lemmas appearing in the text. In this step, we calculate the frequencies of the lemmas for each text. This resulted in a histogram of the lemmas of a certain text.

### **Step 4: Measuring Document-Corpus Discrepancy**

Next, the main question raised above was addressed: *given a pair of documents, what is the discrepancy between them based on the extracted features*. We computed the discrepancy score using the *HC-discrepancy* method described in [18-19, 34]. A low score indicated that it is more likely that the texts were written by the same author, while high values indicated different authorship. Here, a calibration technique for the raw HC-discrepancy scores was developed. The method provides attribution probabilities and appears to enhance the authorship signal.

The HC-discrepancy measure adapts the Donoho-Jin-Tukey Higher Criticism (HC) statistic of [35] to measure the discrepancy between texts using their word-frequency representation (viz. bag-of-words). The method is simple to evaluate and does not involve any parameter tuning. In addition, the HC calculation identifies a subset of discriminating words that are thought to be author characteristics.

In what follows, we review the concept of HC-discrepancy. Later, we use the HC-discrepancy to evaluate if a new text has the same author as any of the existing corpora.

**Notations** Let $T=\{w_{j}{\}}_{j=1,..,M}$ be a document consisting of a set of lemmas. Let us look at a corpus $\mathcal{C=\{}T_{i}{\}}_{i=1,..N}$ as a set of documents, consisting of all the lemmas from all the documents along with their frequencies. We define $\mathcal{C\setminus\{}T\}$ as the corpus $\mathcal{C}$ where the document $T$ is left out, and $\mathcal{C}_{T}\mathcal{=C\cup\{}T\}$ as a new corpus that includes the corpus $\mathcal{C}$ and the document $T$ (the union of $\mathcal{C}$ and the corpus containing only $T$). In addition, $D\mathcal{\in C}$ denotes that the document $T$ is in corpus $\mathcal{C}$, while $D\mathcal{\notin C}$ denotes that the document $D$ is not in corpus $\mathcal{C}$. We also defined $\left| \mathcal{C} \right|$ to be the number of documents in $\mathcal{C}$.

**Definition S1. Higher-Criticism**. Given $N$ p-values $\left\{ p_{i} \right\}_{\left\{ i=1 \right\}}^{N}$, each obtained from a different statistical hypothesis test, the Higher-Criticism measures the global significance of these p-values. It is defined as

$\mathsf{HC}\left( \left\{ p_{i} \right\}_{i=1}^{N} \right)=\max_{1\leq i\leq\gamma_{0}N}\sqrt{N}\frac{\left( \frac{i}{N}-p_{\left( i \right)} \right)}{\sqrt{\frac{i}{N}\times\left( 1-\frac{i}{N} \right)}}$ (1),

where $p_{\left( i \right)}$ denotes the $i$-th $p$-value, sorted in increasing order. Here $\gamma_{0}$ is a tunable parameter that typically has little effect on the large sample behavior of equation (1). We used $\gamma_{0}=0.35$. We also verified that our results are consistent across a wide range of different values of $\gamma_{0}$.

**Remark S1.** In words, HC is obtained by maximizing the difference between the $i$-th $p$-value and its expected value under the uniform distribution, standardized to form a z-score. The version of HC in (1) was proposed in [35], and it provided somewhat better results for the authorship context as opposed to the version of HC defined in [34], see [18] for evaluations using the two versions.

**HC-discrepancy for texts comparison**

In this section, the HC-discrepancy for comparing two texts represented by their word-frequency tables is defined and discussed. Consider a document as an ordered list of words over a prescribed vocabulary $W$. In this paper, we refer to “word” in its broader context as an $n$-gram, i.e., a combination of $n$ consecutive words in the document. For a document $T$ and $w\in W$, denote by $N\left( w|T \right)$ the number of the occurrences of the word $w$ in $T$. The word-frequency table associated with $T$ is the ordered pair $\{\left( w,N\left( w|T \right) \right){\}}_{w\in W}.$

For a given pair of documents, $T_{1}$ and $T_{2}$, we consider $W$ as the set of all words occurring at least once in any of $T_{1}$ and $T_{2}$. For each $w\in W$, we denote

$$n_{w}=N\left( w|T_{1} \right)+N\left( w|T_{2} \right)$$

as the total number of occurrences of the word $w$ in the two documents. Let us also define

$$q_{w}=\frac{\sum_{w'\in W,w'\neq w} N\left( w'|T_{1} \right)}{\sum_{w'\neq w\in W} n_{w'}},$$

An explanation of the motivation behind $q_{w}$, is provided below as part of the hypothesis $H_{0,w}$ description.

Consider the p-value $\pi(w|T_{1},T_{2})$ of the exact binomial test for the hypothesis

$$H_{0,w} : N\left( w|T_{1} \right)\sim Bin\left( n_{w},q_{w} \right),$$

where $\mathrm{Bin}\left( n,p \right)$ is the binomial distribution with $n$ trials and success probability $p$ of each trial.

Roughly,

$\pi(w|T_{1}, T_{2}) := \mathrm{Prob}(|\mathrm{Bin}(n_{w}, q_{w}) - n_{w} q_{w}|) \geq|N(w|T_{1}) -n_{w} q_{w}|)$ (2)

($T_{2}$ is implicitly used in the definition of $q_{w}$)

Note that, due to the properties of the binomial distribution, $\pi(w|T_{1}, T_{2})$ is commutative with respect to the order of $T_{1}$ with $T_{2}$.

The hypothesis $H_{0,w}$ corresponds to the so-called binomial allocation model of $w$ across the two documents, stating that different occurrences of $w$ are independent and each occurrence is equally-likely to originate from $T_{1}$ (respectively $T_{2}$), only accounting for the relative size of $T_{1}$ compared to $T_{1}$ minus occurrences of *w*.

The exact binomial test is applied once per each $w\in W$, result in a collection of p-values

$\Pi\left( W,T_{1},T_{2} \right)=\{\pi\left( w|T_{1},T_{2} \right){\}}_{w\in W}$.

**Definition S2. HC-discrepancy of two documents**. Let $T_{1}$ and $T_{2}$ be a given pair of documents, and let $\pi\left( w|T_{1},T_{2} \right)$ be the p-values obtained for each lemma that exist in the two tested documents as defined in (2). Let us define the HC-discrepancy between $T_{1}$ and $T_{2}$ with the HC score using (1) as

$$d_{\mathsf{HC}}\left( T_{1},T_{2} \right)\mathsf{\equiv HC}\left( \Pi\left( W,T_{1},T_{2} \right) \right).$$

**Remark S2.** We do not rely on the correctness of the underlying binomial allocation model, as there are likely to be deviations due to dependency of some words and other regularities in the text. The HC-discrepancy is merely used here as an index of discrepancy between the two documents in the sense that large values of HC correspond to authorship discrepancy. This use of the HC-discrepancy is known to be effective in resolving authorship challenges even under violations of the binomial allocation model [18]. The correctness of the binomial allocation model is also not necessary in assessing the likelihood of attribution described in Step 5 below.

Next, the definition of the HC-discrepancy is extended in order to compare a document and a corpus of documents $\mathcal{C}$ (where $T\mathcal{\notin C}$), or between two corpora $\mathcal{C}_{1}$ and $\mathcal{C}_{2}$. In both cases, we address a corpus as a long document obtained by concatenating the content of all documents within it.

**Definition S3. HC-discrepancy of two corpora**. Let $\mathcal{C}^{1} =\{T_{i}^{1}{\}}_{i=1,..N}$ and $\mathcal{C}^{2} =\{T_{i}^{2}{\}}_{i=1,..N}$ be a pair of corpora. Let us define $T_{1}=\cup T_{i}^{1}$ and $T_{2}=\cup T_{i}^{2}$ be a new pair of documents, each document is obtained by concatenating all words in each corpus. Using definition S2, the HC-discrepancy between two corpora is defined as

$$d_{\mathsf{HC}}\left( T_{1},T_{2} \right)\mathsf{\equiv HC}\left( \Pi\left( W,T_{1},T_{2} \right) \right).$$

Where $\pi\left( w|T_{1},T_{2} \right)$ be the p-values obtained for each word occurring at least once in any of $\mathcal{C}^{1}$or $\mathcal{C}^{2}$ corpora.

**Definition S4. HC-discrepancy of a document and a corpus**. Let $T$ be a document and $\mathcal{C =\{}T_{i}^{1}{\}}_{i=1,..N}$ be a corpus such that $D\notin\mathcal{C}$. Let us define $T_{1}=\cup T_{i}^{1}$ the document that is obtained by concatenating all documents of $\mathcal{C}$. Using definition S2, the HC-discrepancy between $T$ and $\mathcal{C}$ is defined as

$$d_{\mathsf{HC}}\left( T\mathcal{,C} \right)\mathsf{\equiv HC}\left( \Pi\left( W,T,T_{1} \right) \right).$$

where $\pi\left( w|T,T_{1} \right)$ be the p-values obtained for each word occurring at least once in any of $T$or $T_{1}$.

### **Step 5: Assessing Likelihood of Attribution**

Consider a corpus $\mathcal{C=\{}T_{i}{\}}_{i=1,..N}$ of homogeneous authorship. Let us define the leave-one-out HC-discrepancy score for each document

**Definition S4. Leave-one-out HC-discrepancy score**. Let $\mathcal{C=\{}T_{i}{\}}_{i=1,..N}$ be a corpus, we define the leave-one-out HC-discrepancy score for a document $T_{i}\mathcal{\in C}$ and the $\mathcal{C}$ corpus as

$x_{i}=d_{\mathsf{HC}}\left( T_{i}\mathcal{,C\setminus\{}T_{i}\} \right)$ (3)

Let $X(\mathcal{C})=\{x_{i}\}$ be a set of intra-corpus leave-one-out HC-discrepancies (here $T_{i}$ runs over all the documents in $\mathcal{C}$). In what follows, we assume that the intra-corpus leave-one-out HC-discrepancies $X(\mathcal{C)}$ for a corpus $\mathcal{C}$ of homogenous authorship are independently distributed and follows the same normal distribution. It follows from [18] that HC-discrepancies are usually less affected by topics compared to authorship, implying that the correlation between leave-one-out HC-discrepancies, if exists, is relatively small.

**Attribution Hypothesis testing.** Given a corpus $\mathcal{C=\{}T_{i}{\}}_{i=1,..N}$ and let $T^{'}\mathcal{\notin C}$be a new text, we pose the null-hypothesis $H_{0}$: *the new text and the reference corpus were written by the same author*. In case this hypothesis is true then the extended corpus ${\mathcal{C}_{T^{'}}\mathcal{=C\cup\{}T}^{'}\}$ is of homogenous authorship.

Let us first calculate the HC scores of individual documents in corpus$\mathcal{C}$ with respect to the extended corpus $\mathcal{C'}$

$X\left( \mathcal{C}_{T^{'}} \right)=\left\{ x_{i} \right\}=d_{\mathsf{HC}}\left( T_{i},\mathcal{C}_{T^{'}}\setminus\{T_{i}\} \right)$.

Let us calculate the HC-discrepancy score of the new document with respect to the corpus $\mathcal{C}$ as

$x^{'}=d_{\mathsf{HC}}\left( T^{'}\mathcal{,C} \right)$.

As well as the mean and standard deviation of the HC-discrepancy scores as

$\bar{X}\left( \mathcal{C} \right)=\frac{1}{\left| \mathcal{C} \right|}\sum_{i=1}^{n} x_{i}$, and

$s^{2}=\frac{1}{\left| \mathcal{C} \right|-1}\sum_{i=1}^{n} {\left( x_{i}-\bar{X}(\mathcal{C} \right))}^{2}$ .

Then the t-statistic is defined as

$$t=\frac{x^{'}-\bar{X}\left( \mathcal{C} \right)}{s \sqrt{1+\frac{1}{\left| \mathcal{C} \right|}}},$$

where $\left| \mathcal{C} \right|$ be the number of documents in $\mathcal{C}$.

Under $H_{0}$ and provided $x^{'}$ and $\left\{ x_{i} \right\}$ are independent and follows the same normal distribution, $t$ follows a t-distribution with $\left| \mathcal{C} \right|-1$ degrees of freedom. A p-value ($P$) under $H_{0}$ is the probability of observing a value larger than $t$ assuming $t$-distribution using a table of values from Student's t-distribution. If this event is very unlikely (e.g., $p\leq0.05$), $H_{0}$ can be rejected and we conclude that the new document is $T'$ unlikely to be written by the author of corpus $\mathcal{C}$.

We note that it is possible to carry over (and we initially did) a similar analysis using a non-parametric test as in [18]. Nevertheless, we preferred the parametric Gaussian model because it is more robust in our context as bootstrap analysis implies.

### **Step 6: Attributing Authorship**

In this subsection, we discuss the attribution process itself. Given a document $T'$ of unknown authorship and $m$ references corpora $\left\{ C_{j} \right\}_{j=1,..,m}$, each associated with a different candidate author. Noteworthy, that our analysis uses the same p-values (Step 5 of the method) for two distinct tasks: (1) hypothesis testing with respect to individual authors, i.e. authorship verification. (2) Deciding on the most likely author among the candidates, i.e. authorship attribution. In task (1), failure to reject the null hypothesis does not necessarily mean that the null hypothesis is true. It simply means that the evidence that we currently have is insufficient. It is very possible that we reject the same null given more data is available. In the context of our analysis, this means that we cannot rule out the possibility that the examined text was not written by the author against which we are testing. In task (2), we associate the text to whichever author has it most likely to be the author under the probabilistic model we derive in Step 5. It happens to be so that under the model assumptions, the most likely author also has the largest p-value.

We perform pair wise authorship attribution, and attribute the text to the most likely corpus. First, the HC-discrepancies of $T'$ is calculated with respect to each with each of the reference corpora, this process results in $x_{j}$. Next, the p-values ($p_{j})$of associating document $T'$ with respect to the $m$ reference corpora are estimated using the t-distribution, where, $p_{j}\in\left[ 0,1 \right]$. Later, the new text is associated with the corpus that has the largest p-value:

$j^{*}=\text{argmax}_{j=1,\ldots,m}p_{j}$.

We conclude that the current text was most likely written by the author $j^{*}$. Nevertheless, if $p_{j^{*}}$ is too small, we state that the probability for the attribution is small, and we may conclude that neither of our candidate authors wrote $D'$.

### **Step 7: Reasoning Attribution Procedure**

In order to identify the discriminating words, we follow the Higher Criticism Thresholding (HCT) procedure proposed in [43] The HC calculation in equation [(1)](#eq:HC:def) considers the maximum of z-scores $z_{i}=\left( i/N-p_{\left( i \right)} \right)/\sqrt{i/N\left( 1-i/N \right)}$. We maximize these z-scores over the range $0<i\leq\gamma_{0}N$ and denote the maximal index by $i^{*}$. The set of p-values selected in the HCT procedure is

$$\Delta^{*}\left( \{p_{i}{\}}_{i=1}^{N} \right)\equiv\{p_{\left( 1 \right)},\ldots,p_{\left( i^{*} \right)}\}.$$

Intuitively, HCT selects a set of the smallest p-values that derive the largest deviation of the standardized empirical process represented by the ${\{z}_{i}\}$ from the uniform empirical process which arises when the p-values are uniform. In practice, we think of words or n-gram whose p-value is included in $\Delta^{*}\left( \{p_{i}{\}}_{i=1}^{N} \right)$ as features providing the best evidence against authorship similarity of the two documents.

# Experiment Details and Results

## 3.1 Ground-truth chapter attribution

Our experiment was conducted on biblical texts that contained 50 texts each accompanied with corpora attribution, specified by the biblical scholar in our team. First, we analyzed the results on the ground-truth dataset. Later, we applied our methodology to a group of texts for which there is no consensus between biblical scholars regarding their attribution. Following are the specifications and the results of our experiments.

We choose biblical texts that pertain to one of the three corpora: Deuteronomy, Deuteronomistic History, and Priestly materials, where their chapters are listed in the main text. In addition to these 50 chapters, in order to deal with the same genre, we opted for several narration chapters to enhance the P corpus. As no clear-cut P chapters could be taken as narration, to complete our dataset we decided to add P verses (to differ from chapters) from Genesis and Exodus. These were used only as reference data for the HC calculation and were not tested for author attribution (since they were too short to be securely attributed). The following versus were added to the Priestly material: Gen 1:1-31, Gen 2:1-3, Gen 5:3-28,30-32, Gen 6:9-22, Gen 9:1-17,28-29, Gen 10:2-7,20,22-23,31, Gen 11:11-26,29-32, Gen 12:5, Gen 13:6,12, Gen 16:3,15-16, Gen 21:2-5, Gen 22:20-24, Gen 23:1-20, Gen 25:7-10,13-17,20, Gen 26:20,34-35, Gen 27:46, Gen 28:1-9, Gen 35:9-15,27-29, Gen 36:40-43, Gen 37:1, Gen 46:6-7, Gen 47:28, Gen 49:29-33, Gen 50:12-13, Exod 1:1-4,7,13-14, Exod 2:23-25, Exod 7:1-13,19-22, Exod 8:1-3,11-15, Exod 9:8-12, Exod 11:9-10, Exod 12:40-42, Exod 13:20, Exod 14:1-4,8-10,15-18,21-23,27-29, Exod 15:22, Exod 19:1, Exod 24:16-17, where the comma separates different verses in the same chapter.

We provide herein some information regarding the lengths of these texts, which as will be later discussed is tightly related to the expected accuracy. There are nine Deuteronomy texts, that consist of 6324 lemmas, with 544 median number of lemmas per chapter; 19 Deuteronomistic History chapters that consist of 13410 lemmas, with 574 median number of lemmas per chapter; and 22 Priestly texts with 14560 lemmas (and 5077 lemmas more in the auxiliary data), with 691 median number of lemmas per chapter. In total, our ground truth dataset consists of 50 chapters and 34294 lemmas. See also Table S1-S2 and Fig. S1 in the SI for additional information.

**Table S1.** **The lengths of the texts in our dataset.**

| **Author** | **Chapter** | **Num. Words** | **Num. Verses** |  | **Author** | **Chapter** | **Num. Words** | **Num. Verses** |  | **Author** | **Chapter** | **Num. Words** | **Num. Verses** |
| --- | --- | --- | --- | --- | --- | --- | --- | --- | --- | --- | --- | --- | --- |
| D | Deut.6 | 540 | 25 |  | DtrH | Deut.8 | 474 | 20 |  | P | Exod.16 | 825 | 36 |
| D | Deut.12 | 855 | 31 |  | DtrH | Deut.9 | 810 | 29 |  | P | Exod.25 | 710 | 40 |
| D | Deut.13 | 538 | 19 |  | DtrH | Deut.10 | 530 | 22 |  | P | Exod.26 | 741 | 37 |
| D | Deut.15 | 590 | 23 |  | DtrH | Deut.11 | 837 | 32 |  | P | Exod.27 | 411 | 21 |
| D | Deut.16 | 536 | 22 |  | DtrH | Deut.27 | 483 | 26 |  | P | Exod.28 | 913 | 43 |
| D | Deut.18 | 480 | 22 |  | DtrH | 1Kgs.8 | 1797 | 66 |  | P | Exod.29 | 1071 | 46 |
| D | Deut.19 | 544 | 21 |  | DtrH | 2Kgs.17 | 530 | 41 |  | P | Exod.30 | 740 | 38 |
| D | Deut.26 | 568 | 19 |  | DtrH | 2Kgs.22 | 574 | 20 |  | P | Exod.31 | 362 | 18 |
| D | Deut.28 | 1673 | 69 |  | DtrH | 2Kgs.23 | 1177 | 37 |  | P | Exod.35 | 696 | 35 |
|  |  |  |  |  | DtrH | 2Kgs.24 | 466 | 20 |  | P | Exod.36 | 779 | 38 |
|  |  |  |  |  | DtrH | 2Kgs.25 | 777 | 30 |  | P | Exod.37 | 590 | 29 |
|  |  |  |  |  | DtrH | 2Sam.7 | 745 | 29 |  | P | Exod.38 | 645 | 31 |
|  |  |  |  |  | DtrH | Josh.1 | 494 | 18 |  | P | Exod.39 | 855 | 43 |
|  |  |  |  |  | DtrH | Josh.12 | 349 | 24 |  | P | Exod.40 | 686 | 38 |
|  |  |  |  |  | DtrH | Josh.23 | 518 | 16 |  | P | Exod.6 | 598 | 30 |
|  |  |  |  |  | DtrH | Josh.5 | 448 | 15 |  | P | Gen.17 | 575 | 27 |
|  |  |  |  |  | DtrH | Josh.6 | 754 | 27 |  | P | Lev.1 | 399 | 17 |
|  |  |  |  |  | DtrH | Judg.2 | 574 | 23 |  | P | Lev.2 | 307 | 16 |
|  |  |  |  |  | DtrH | Judg.6 | 1073 | 40 |  | P | Lev.3 | 388 | 17 |
|  |  |  |  |  |  |  |  |  |  | P | Lev.4 | 840 | 35 |
|  |  |  |  |  |  |  |  |  |  | P | Lev.8 | 902 | 36 |
|  |  |  |  |  |  |  |  |  |  | P | Lev.9 | 527 | 24 |
|  |  |  |  |  |  |  |  |  |  |  |  |  |  |

**Table S2.** **Ground truth test length statistics**

|  | D | DtrH | P | Total |
| --- | --- | --- | --- | --- |
| Num. of Chapters | 9 | 19 | 22 | 50 |
| Shortest chapter (verses) | 19 | 15 | 16 | 15 |
| Longest chapter (verses) | 69 | 66 | 46 | 69 |
| Median num. of verses | 22 | 25 | 35 | 28 |
| Total num. of lemmas | 6324 | 13410 | 14560 | 34294 |
| Median num. of lemmas | 544 | 574 | 691 | 590 |

We assume that each corpus represents an author or authors of the same cultural/ideological background. The length of these texts appears in Table [S1](#Text_Lengths)-S2 and Fig. S1. As can be seen in the data provided in the main text and Table. S1, the texts are not very long (the shortest contained around 300 words, the longest 1800 words, and the median length was 590 non-unique words). As can also be seen in Figure S1 the length of the texts of all corpora are approximately of the same magnitude, where the majority of the texts range between 300-900 words, with a few more texts with a little bit more information (900-1800 in length). Although, corpus P had substantially more information.


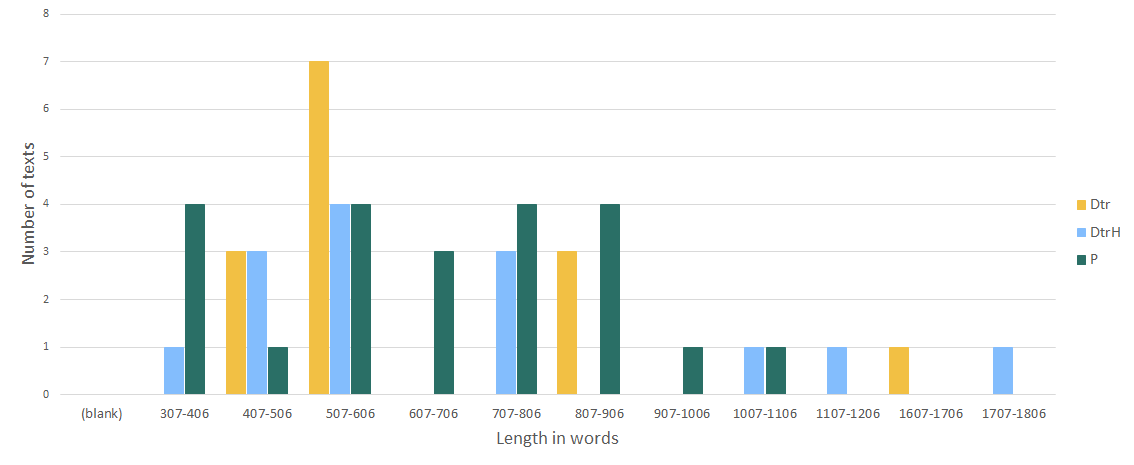


**Figure S1.** **A histogram plot of the lengths of the text of the three corpora**: D (yellow) DtrH (blue), and P (pine green).

The texts in this study were selected because they contained enough statistical information, are considered to be minimally edited and can be attribution can be verified versus one of the three corpora. Thus, the writing characteristics of each writer can be extracted and used for the attribution task of new texts.

Deuteronomistic historiography (DtrH) relies on the Deuteronomic code (D) to understand and explain the history of the kings of Israel. It is the observance and non-observance of the laws that makes it possible to classify the kings into good and bad kings and to explain the destruction of Samaria and later of Jerusalem. If these two corpora are close in their themes, there is however a debate as to whether D and DtrH were composed by the same circles under Josiah (English-speaking school) or whether DtrH is a later composition to explain the exile (German school) by a similar theological milieu. As for the P-texts, they should easily be distinguished from the other two corpora. On the one hand, these texts date from the end of the exile or from the post-exilic era and propose another reading of history: inclusive and universal. The importance of rites, sanctuary, and clergy is another typical element.

The DtrH and P corpora have in common that they are redactions that are found in several different biblical books. We performed a leave-one-out procedure and calculated the HC value of each text with respect to the three existing corpora. The HC calculation in equation [(3)](#eq:HC:def) involves computing the probability for each word and finding the words with the smallest probability. We will illustrate the HC calculation on two examples, Deut 26 (D corpus) and Deut 11 (DtrH corpus).

In Figure [S2](#fig:Discriminating_words_Example_1) the discriminating lemmas of Deut 26 with respect to the three corpora are listed. Each graph in Figure [S2](#fig:Separating_words) shows a list of at most 20 of the discriminating lemmas for each of the three corpora vs the union of the other two. The lemmas are ordered by their significance level (log of their p-value). The sign of score indicates whether the word had high frequency (appeared more) in the current corpus or in the one we are comparing against (positive or negative respectively). As can be seen, Deut 26 has common lemmas: ו, ל (to, and) with D, as ל,ו, אלוהי, נתן, (*ntn*, to give, *ʾᵉlōhim*, God, *w*, and, *l*, to) with DtrH and ל, אלהים, מצוה, קול (*ʾᵉlōhim*, God, *l,* to, *qôl*, voice, *miṣwâh*, commandment) with P. The HC scores are 0.43, 1.9, 3.61 for the D, DtrH, and P corpus respectively. Thus, the minimal value of 0.43 implies that we attribute Deut 26 to D, the attribution chosen by the biblical scholar in our team.


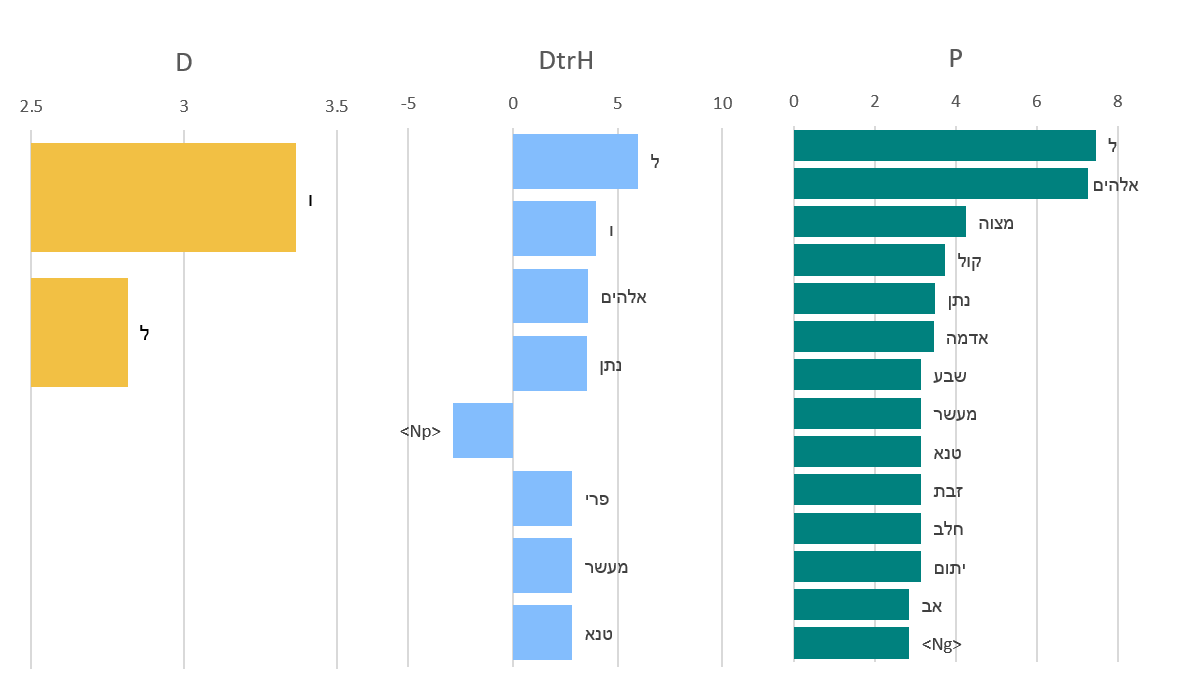


**Figure S2.** **Discriminating lemmas of Deut 26 with respect to each corpus** are presented in three graphs (left to right: D, DtrH, P). Each graph lists at most the top 20 lemmas that are the most important for distinguishing Deut 26 from the three corpora. The lemmas were selected by the HC algorithm and are ordered by their significance level (log of the p-value).

Another example is shown in Figure S3, where we plot the discriminating lemmas of Deut 11. Although this chapter is attributed to DtrH by the experts in our team, its word statistic resembles the D more; this is reflected in the HC scores: -0.9, 1.88, 4.59, for the D, DtrH, and P corpus respectively. We can examine the discriminating lemmas of Deut 11 to understand the reason for its attribution to D. We see that Deut 11 has the following common words with D את, ירש, יום, (*yôm*, day, *yrš*, to possess, *ʾet*, direct object particle) while with DtrH it has ירש, ברכה, אנכי, מצוה and <Np>, (*miṣwâh*, commandment, *ʾānōki*, I, *bᵉrākâh*, blessing, *yrš*, to possess) and with P its ירש, אנכי, אשר, מצוה, שמר (*šmr*, to keep, *miṣwâh*, commandment, *ʾᵃšer*, which, *ʾānōki*, I, *yrš*, to possess) This tool enables scholars to examine the attribution, and verify the reason for the HC attribution.


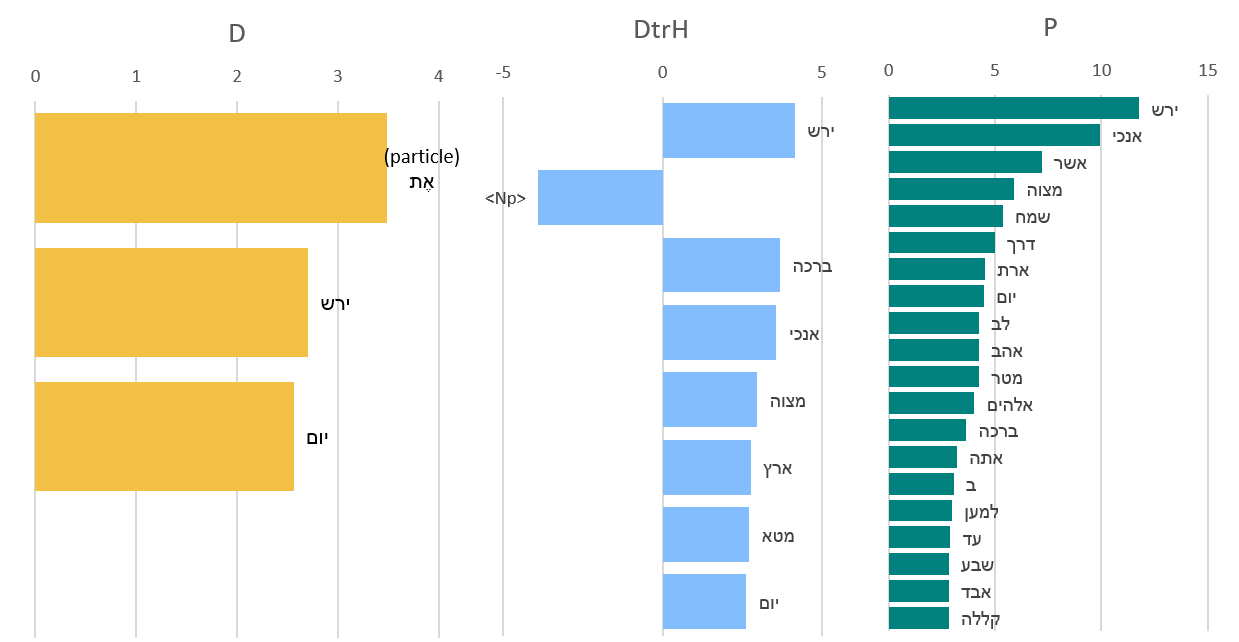


**Figure S3. Discriminating lemmas of Deut 11 with respect to each corpus** are presented in three graphs (left to right: D, DtrH, P). Each graph lists at most the top 20 lemmas that are the most important for distinguishing Deut 11 from the three corpora. The lemmas were selected by the HC algorithm and are ordered by their significance level (log of the p-value). Noteworthy that Deut 11 was attributed to D by the algorithm, rather than to DtrH as expected. The words used to reject the same-author hypothesis with DtrH were ירש, <Np>, ברכה, אנכי, מצוה, ארץ, מטר, .יום

All the results of the HC score are summarized in Table S3 using the same notations as in Table [1](#fig:t_pval_values) of the main text. The examined chapter names head the columns of the table, while the potential corpora name heads the rows, with the intersection cells providing the p-values. The cell containing the chapter name is colored according to the ground-truth attribution (yellow for D, blue for DtrH, and pine green for P). Attributing the texts based on the minimal HC value with respect to the three corpora (minimal value in each row) our accuracy is $84\%$. The attributions that do not coincide with biblical expert attribution are Deut 13, 15 attributed to DtrH instead of D, Deut 8, 11, Judg 2 attributed to D instead of DtrH, Josh 5, 23 attributed to P instead of DtrH, and Exod 16; attributed to DtrH instead of P.

**Table S3.** **HC values of the ground-truth texts.**

The table presents the HC values of each text with respect to the three reference corpora (as opposed to Table 2 where p-values are presented). Each text can be attributed to one of D, DtrH, or P based on the min HC score (marked orange) between the text to each corpus in a leave-one-out fashion.

The HC data is also presented in a graphical manner in Figure [S4](#fig:HC_in_2D) where each panel shows a pairwise comparison of two corpora attribution based on their HC score. The points are colored according to their predefined lab (Panel A shows a comparison of D vs DtrH. Panel B shows D vs P, and panel C DtrH vs P). The ground-truth corpora can be clearly seen as a cluster, with only a few misclassifications. For instance, the HC values of the D corpus appear as a cluster in (A), and (B), the same goes for the other two corpora. This HC similarity is the base for the attribution and for calculating the likelihood of the attribution by means of estimating the distribution of each corpus.

All the chapters, whose HC score is to the left/right of the (x=y) line, have a smaller/larger value in the vertical HC coordinate, compared to the horizontal coordinate, showing the preferred attribution. This is a trivial clustering criterion, and as will be seen later, modeling the distribution of each corpus will give more robust and accurate results.


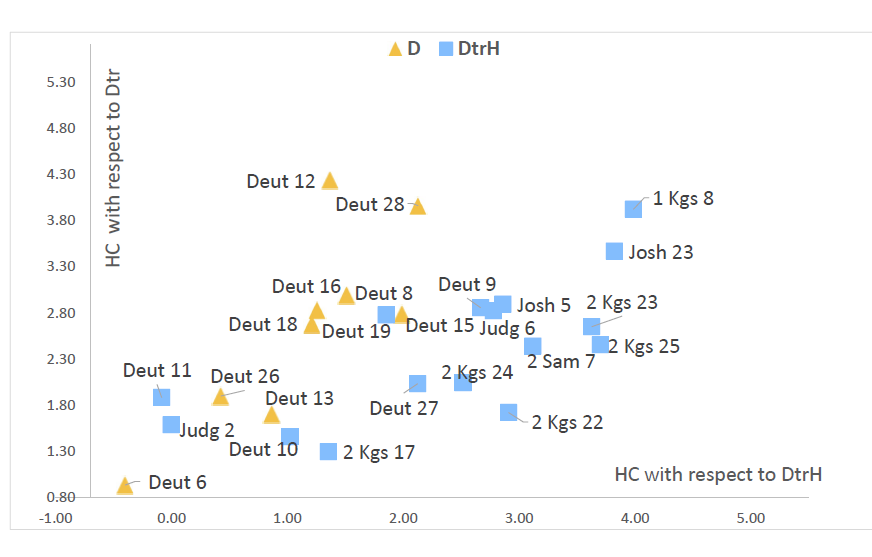


**(A)**


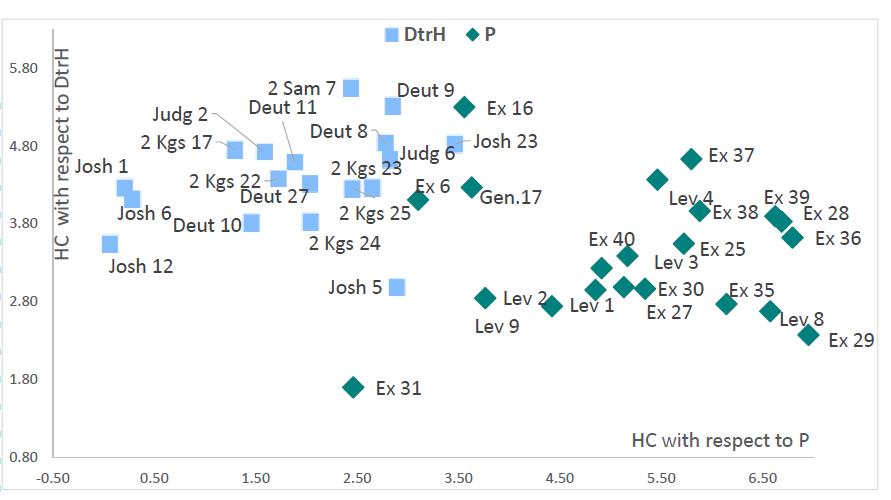


**(B)**


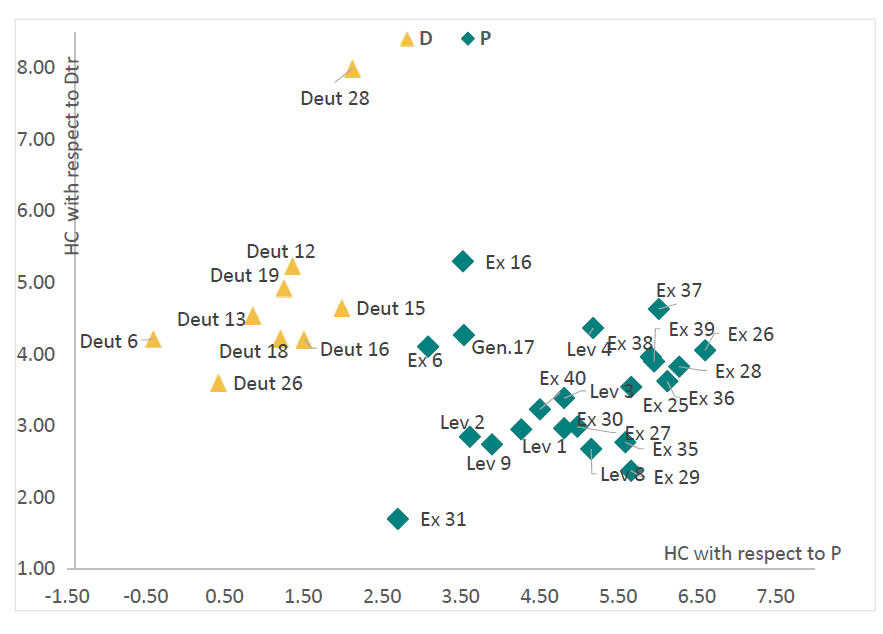


**(C)**

**Figure S4. Pairwise comparison of two ground-truth corpora based on their HC values**. **(A)** present the comparison of D vs DtrH, **(B)** D vs P, and **(C)** DtrH vs P (yellow triangle for D, blue squares for DtrH, and pine green rhombus for P).

Following the HC score calculation, we turn to estimate the distribution of the HC scores of each corpus, which results in the median and variance parameters for each corpus. Next, the probability for attributing each text to one of the reference corpora is calculated. These probabilities are presented in Table [1](#fig:t_pval_values) in the main text. As mentioned above, p-values of less than $0.05$ are colored in blue, meaning that it is not likely that the current chapter can be attributed to the corresponding corpora. As can be seen, the False Negative (FN) rate is very low and is only $4\%$ (see Table S4 for additional details). This low FN rate indicates that our attribution method describes well the existing corpus and that if we conclude that it is unlikely that a certain author wrote a text it is with high accuracy. In addition, we observe that almost the entire corpus P is distinct, and cannot be attributed to either D or DtrH, since the p-values are less than the selected threshold.

**Table S4. The accuracy of attribution likelihood assessment**

| n=50 | Negative | FN=0 | TN=19 |  | Negative | FN=1 | TN=18 |  | Negative | FN=1 | TN=4 |
| --- | --- | --- | --- | --- | --- | --- | --- | --- | --- | --- | --- |
|  |  | **D Corpus** | |  |  | **DtrH Corpus** | |  |  | **P Corpus** | |

Next, we turn to the attribution task itself. It should be noted that although attribution of a given text could be performed by selecting the minimal HC score with respect to the three corpora, we calculated the probability of the attribution using the t-distribution of the entire corpus. Our experiments showed that this results in a robust attribution algorithm that takes into account the given corpora data. As can be seen from Table [1](#fig:t_pval_values), selecting the maximal value for each text results in $84\%$ accuracy. The eight chapters for which the two do not agree are as follows: Deut 13, and 15 were automatically attributed to DtrH instead of D. These texts may have undergone dtr revision. (b) Deut 8, 11, and Judg 2 were attributed to D instead of DtrH. The differences between D and DtrH are in fact not so important, and both corpora share a significant vocabulary. (c) Josh 5, and 23 were attributed to P instead of DtrH. Josh 5 indeed contains passages that are inspired by priestly terms and language. For Josh 23 this attribution is mysterious since it is by all specialists considered to be “dtr.” (d) Exod 16 was attributed to D instead of P. This can be explained by the fact that the P text in Exod 16 was revised later in a Deuteronomistic language.

In Tables S4-6 we present a more detailed analysis of the attribution task. These results demonstrate the soundness of our algorithmic sequence. For brevity, we will use the following notation true positives (TP), true negatives (TN), false positives (FP), and false negatives (FN). In Table S5 we present a normalized multi-class attribution confusion matrix for the author attribution task. This table was constructed by assessing the performance of the proposed method with respect to the three corpora, and later normalized with respect to the entire true class. As can be seen, the TP values of the diagonal elements represent the degree of correctly predicted classes. Although the D corpus has only 9 texts performs fairly well, with 78% success rate. Also, we see that the richness of the P corpus, as well as its distinctiveness from the other two result in an attribution TP of 95%. The confusion is expressed by the false classified off-diagonal elements since they are mistakenly confused with another class. Due to the adherence of the D and DtrH corpus, there are 3 DtrH chapters that were wrongly attributed to D, and 2 texts from D attributed to DtrH.

**Table S5.** **Normalized confusion matrix for a multi-class attribution.**

|  | True class | | | |
| --- | --- | --- | --- | --- |
| Predicted | % | D | DtrH | P |
|  | D | 0.78 | 0.17 | 0.05 |
|  | DtrH | 0.22 | 0.72 | 0 |
|  | P | 0 | 0.11 | 0.95 |

The cells' background corresponds to their value (green for high values, and red for small).

A detailed analysis of binary classification accuracy is provided in Table S6 for each of the three corpora. For each corpus, we assessed the performance of the proposed method and again normalized each column with respect to the true total value. Note that the tables in Table S6 are not just a rearrangement of the normalized confusion matrix for a multi-class attribution. The new information that we see here is the high TN rate (90%, 94%, and 93%), meaning that with high accuracy we do not mistakenly associate texts (even for a fairly small corpus like D). We also note that our FP is rather low for the three corpora.

**Table S6. Binary-class attribution confusion matrix (presented in percental) for each corpus (D, DtrH, P).**

|  |  | True class | | |  | |  | | True class | |  |  | True class | |
| --- | --- | --- | --- | --- | --- | --- | --- | --- | --- | --- | --- | --- | --- | --- |
| Predicted | % | Positive | Negative |  | | % | | Positive | | Negative |  | % | Positive | Negative |
|  | Positive | TP=0.78 | FP = 0.1 |  | | Positive | | TP=0.72 | | FP = 0.06 |  | Positive | TP=0.95 | FP = 0.07 |
|  | Negative | FN=0.22 | TN=0.9 |  | | Negative | | FN=0.28 | | TN=0.94 |  | Negative | FN=0.05 | TN=0.93 |
|  |  | **D Corpus** | | |  | |  | | **DtrH Corpus** | |  |  | **P Corpus** | |

Last, we calculated the accuracy measured using precision, recall, and f1-scores using the confusion matrices (Table S7). The idea here is to report on the precision/recall/f1 of every class as if we have a binary classifier for that class vs. the others. The high value of the reported measures demonstrates the soundness of our algorithmic apparatus.

**Table S7. Corpus-wise attribution accuracy measured using precision, recall, and f1-scores.**

| Corpus | Precision | Recall | F1 - score |
| --- | --- | --- | --- |
| D | 0.89 | 0.78 | 0.83 |
| DtrH | 0.92 | 0.72 | 0.81 |
| P | 0.93 | 0.95 | 0.94 |

## Other Biblical Texts

In what follows the same process was performed on the other texts for which there is no agreement about attribution among biblical scholars. The texts that were tested are: Deut 4, Lev 26, Ark Narrative 1, Ark Narrative 2, Chronicles 1 and Chronicles 2, Late Abraham material, the Gibeah story, the early Jacob story, Esther, and Proverbs wisdom literature. The chapter numbers as well as the verses of these texts are listed in the main text and the lengths of the texts were compared to the ground-truth corpora texts shown in Figure S5. As can be seen, except for Ark 2 and Chr 2, most of the texts are much longer than the ground-truth texts, and they contain a great deal of statistical information. This fact implies that the attribution process itself will result in more reliable results (as discussed in the Accuracy and Robustness Evaluation subsection).


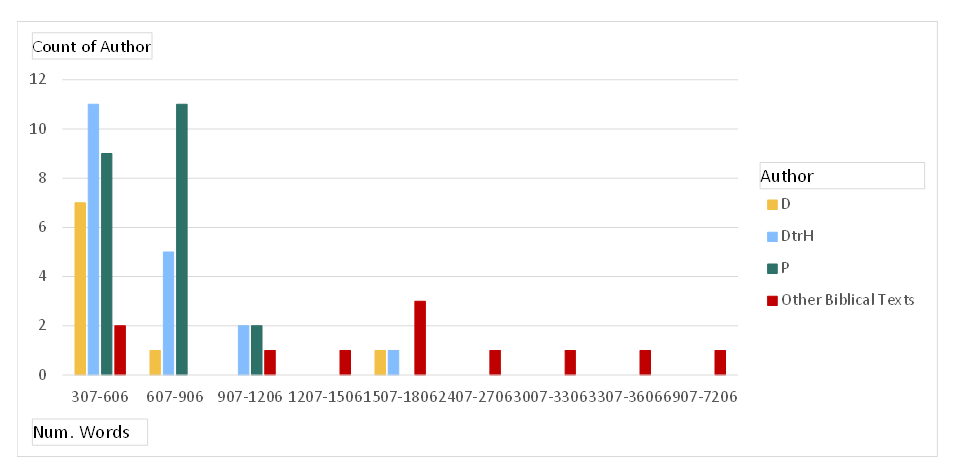


**Figure S5.** **The texts lengths of the three corpora and the other biblical texts discussed**. D is colored in yellow, DtrH in blue, P in pine green, and the other biblical texts in red.

Next, the HC values for each new biblical text were calculated with respect to the three ground-truth corpora. The values are listed in Table S[8](#fig:Unk_HC_values), where the minimal HC score for each text is marked in orange. Even though we do not attribute the texts based on their HC score, it is interesting to compare the HC scores, and their scale to the HC scores of the ground-truth corpora. In Figure S6 the HC values of these texts with respect to the three corpora are shown. For convenience, the Prov and Chr texts are omitted from the graph since their HC values are not in the scale with the other HC values. For the HC values with respect to the D and DtrH, we see that late Abraham and Gibeah are far from the existing corpora (and also Prov and Chr). In addition, for DtrH Lev 26, Ark 1, and Esther. the likelihood of being attributed to D or P is very small). In the D and P comparison, we see that aside from late Abraham and Gibeah, Esther, Early Jacob are also far from the ground-truth corpora, and Deut 4, Ark 1, and Lev 26 have a very small likelihood of being attributed to D or P. It should be emphasized that although in Figure S6 (B) we see that Abraham and Gibeah and also Esther and Early Jacob appear close to each other, we cannot conclude that they were written by the same author (i.e., belong to a fourth author). However, this shows that HC calculations give a great deal of weight to the literary genre as all of these stories are narratives and HC calculations are not always able to distinguish redactional milieux.

**Table S8.** **The HC values of the disputed texts.**

The table presents the HC values of each text with respect to the three reference corpora. Each text can be associated with one of D, DtrH, or P based on the min HC score between the text to each corpus in a leave-one-out fashion. The minimum HC score in each column is marked in orange, associating the chapter with the corresponding corpus.

In some cases, the new tested texts are closer to the existing clusters, but in order to test *how close they are*, we rely on the distribution of the HC scores of the three corpora. The mean and standard deviation are approximated for each of the ground-truth corpora, and the p-values for the attribution of the new texts are calculated. For example, we pose the null hypothesis that *Chr was written by the same author who wrote the D corpus* and received a p-value of 6.400E-04. Since these p-values are less than our threshold of 0.05, we negate the null hypothesis, and conclude the Chr was not written by the author of the D corpus. This process is repeated for the DtrH and P corpora, which results in 5.197E-06, 4.957E-08 values. Next, the null hypothesis is posed for the other biblical texts, and summarize the results in Table 2 in the main text. As can be seen the texts Lev 26, Ark 1, Chr, the late Abraham material, the Gibeah story, and the Proverbs literature are unlikely to be written by either of the three. This result supports the intuition obtained from Figure S6. Deut 4, the early Jacob story are unlikely to be associated with P. Esther cannot be associated with P.


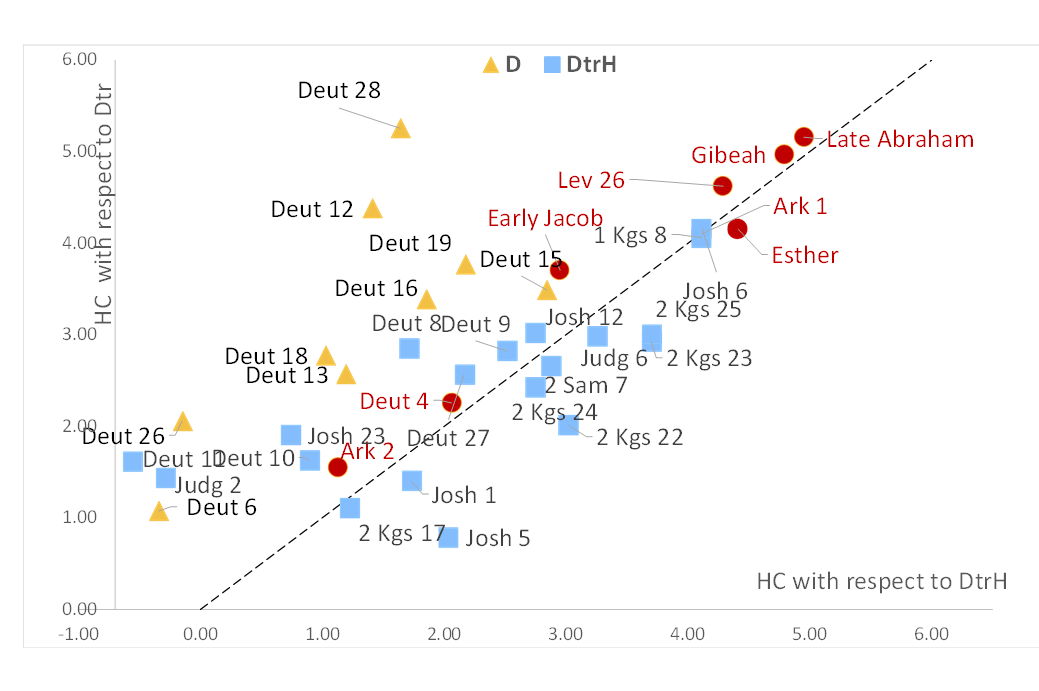


**(A)**


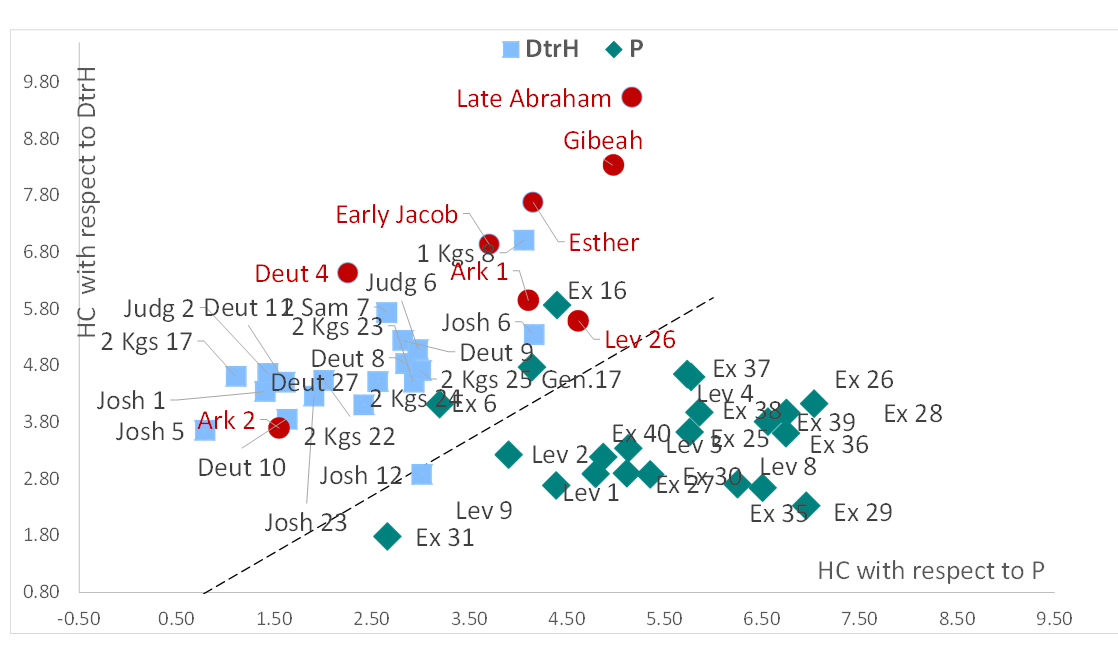


**(B)**


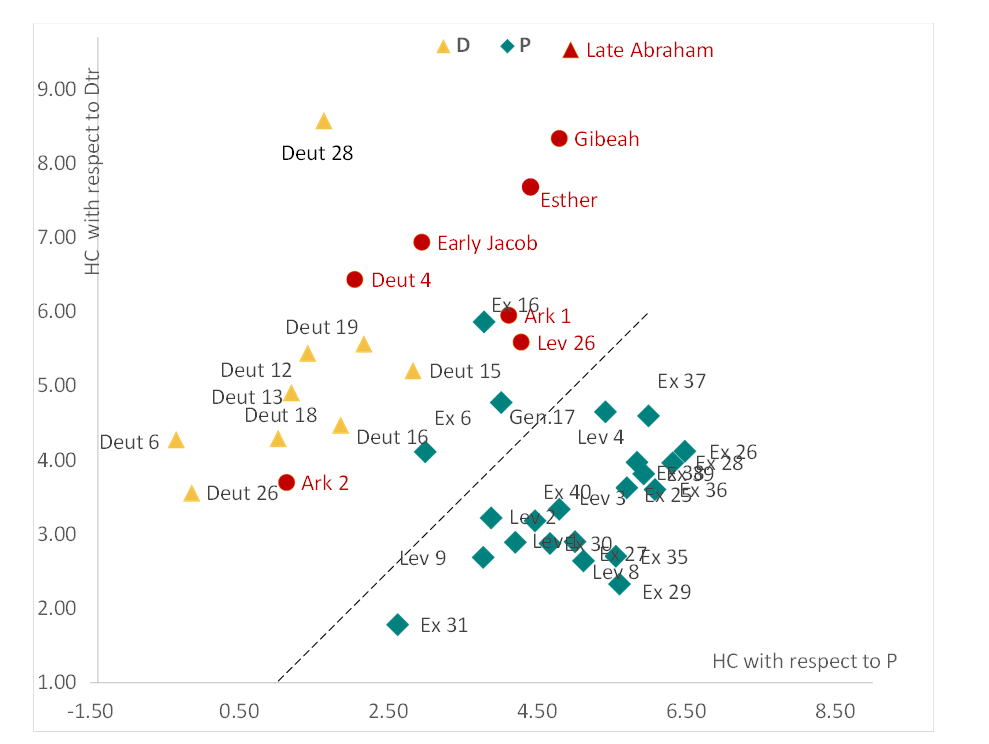


**(C)**

**Figure S6**. **Pairwise comparison of two ground-truth corpora based on their HC values**. **(A)** the comparison of D vs DtrH is presented, **(B)** DtrH vs P, **(C)** D vs P. Yellow triangle represents D, blue squares DtrH, pine green rhombus P, and red circles for the other biblical texts).

From the graphs in Figure S6, it is hard to see which of the three corpora better fits a given text. The estimated attribution probability is used to deal with this task. The corpus which is the most likely to be written by the same author as a given text is selected. In Table 2 the highest p-value score for each tested text is selected. From the previous step, we already know that Lev 26, Ark 1, Chr, the late Abraham material, the Gibeah story, and the Proverbs literature cannot be associated with either of the three ground-truth corpora. The text in Deut 4 is more likely to be attributed to DtrH. Ark 2 is more likely to be attributed to DtrH. All the results are summarized in Table S9. In Figure S7 we present the lists of the discriminating lemmas of all the other biblical texts.

**Table S9**: **Summary of the association of the additional texts**.

|  | Same author hypothesis was rejected for this corpus. Cannot be associated with | May be associated with | A set of “discriminating words” which have the biggest effect on the value of the HC statistic in rejecting same author hypothesis  (-) – indicate negative affect |
| --- | --- | --- | --- |
| Deut 4 | P | DtrH | תבנית, למד, חק, אש, אלהים, פן, שמים  *šāmayim*, heavens; *pen*, lest; *ʾᵉlōhim*, God; *ʾēš*, fire; ̣ *ḥōq*, statute; *lmd*, to learn; *tabnit*, pattern |
| Lev 26 | All three |  | N\A |
| Chr | All three |  | N\A |
| Early Jacob | P | D (13% prob. For association) | מלך, ה, אלהים, <Np>, אשה, אמר, ארץ (-), סריס  *melek*, king; *h*, the; *ʾᵉlōhim*, God; *ʾiššâ*, woman; *ʾmr*, to say; *ʾereṣ*, earth; *sāris*, eunuch |
| Late Abraham | All three |  | N\A |
| Esther | P, and most likely not DtrH nor D (only 6 and 5 % prob. For association) |  | N\A |
| Proverbs | All three |  | N\A |
| Ark Narrative 1 | All three |  | N\A |
| Ark Narrative 2 |  | DtrH | ארון, <Np>, בית, ברך, אמה, כבד  *ʾᵃrôn*, ark; *bayit*, house; *brk*, to bless; *ʾāmâh*, handmaid; *kbd*, to honor |
| Gibeah story | All three |  | N\A |

| 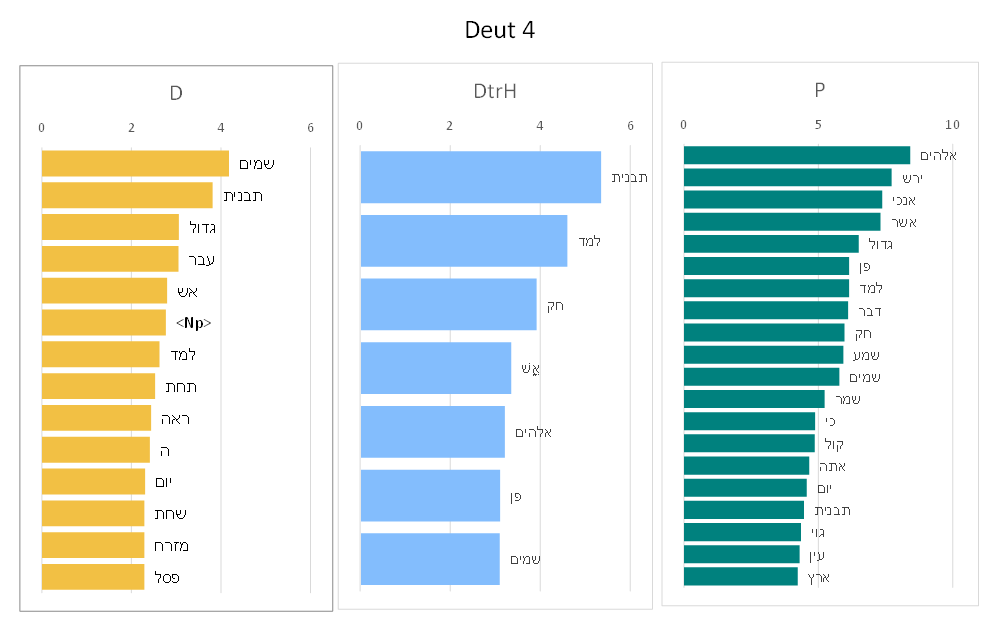  (A) | 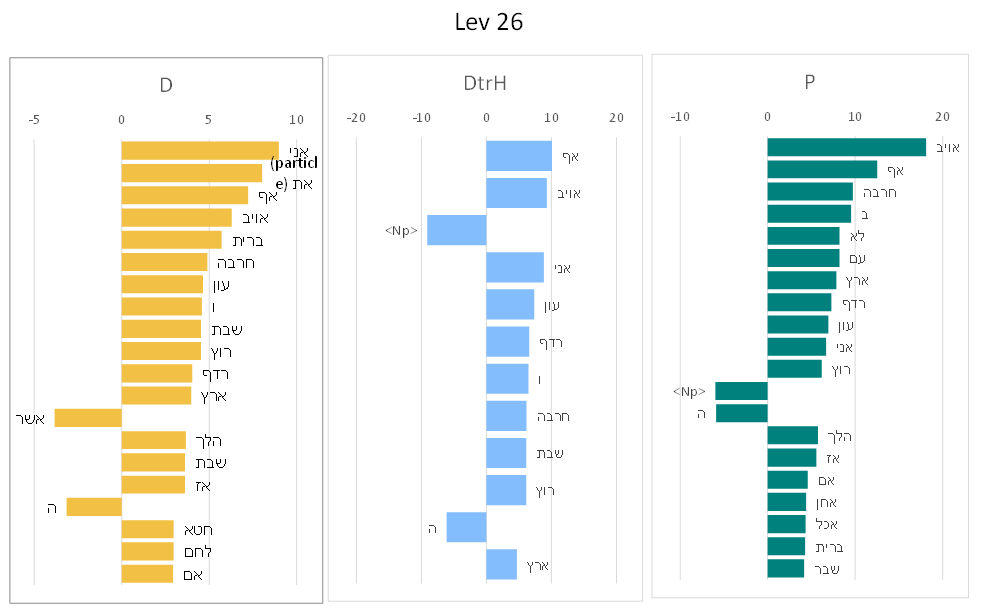  (B) |
| --- | --- |
| 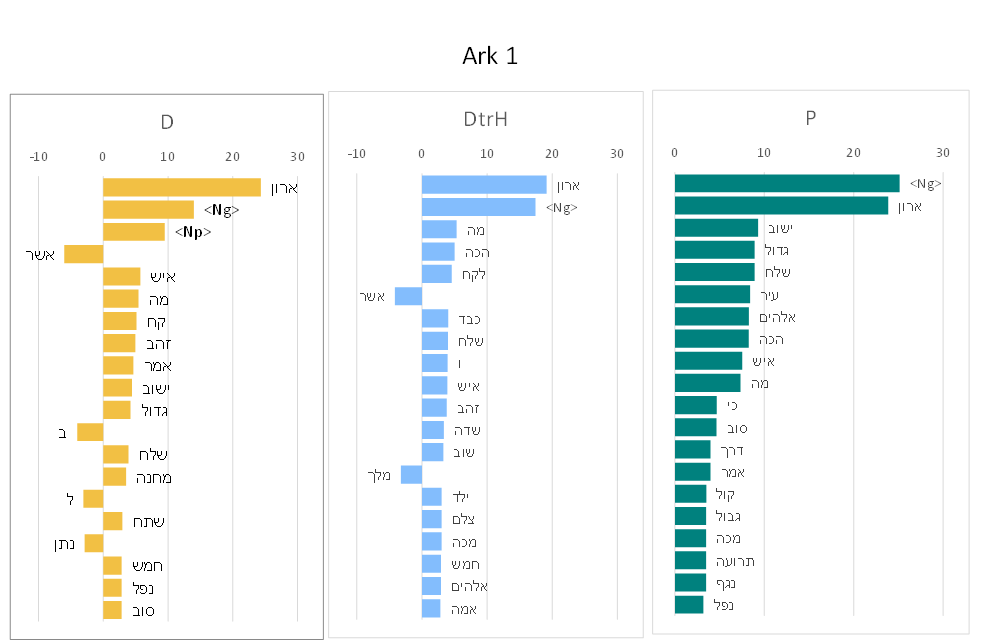  (C) | 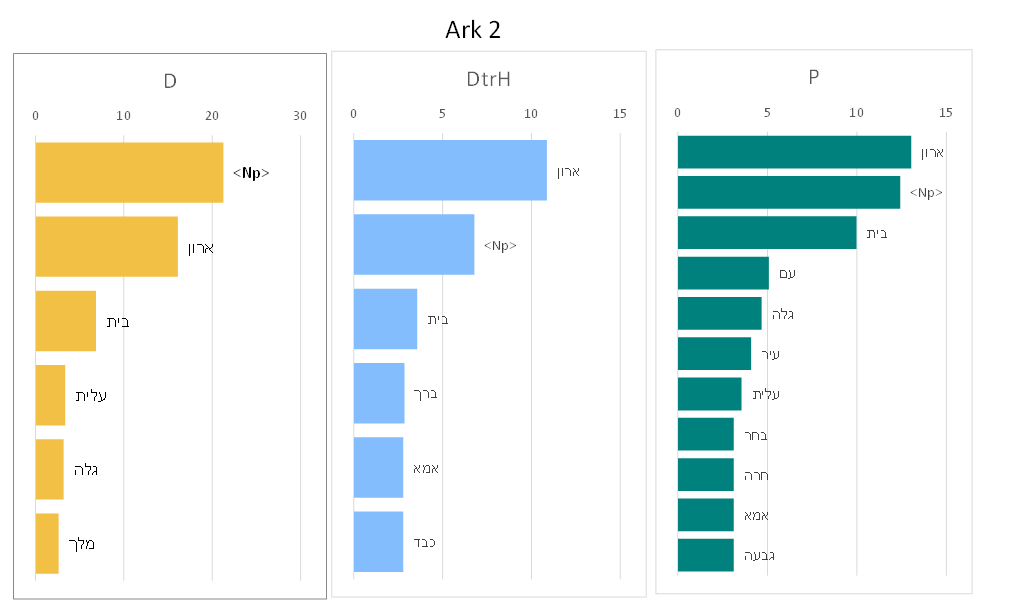  (D) |
| 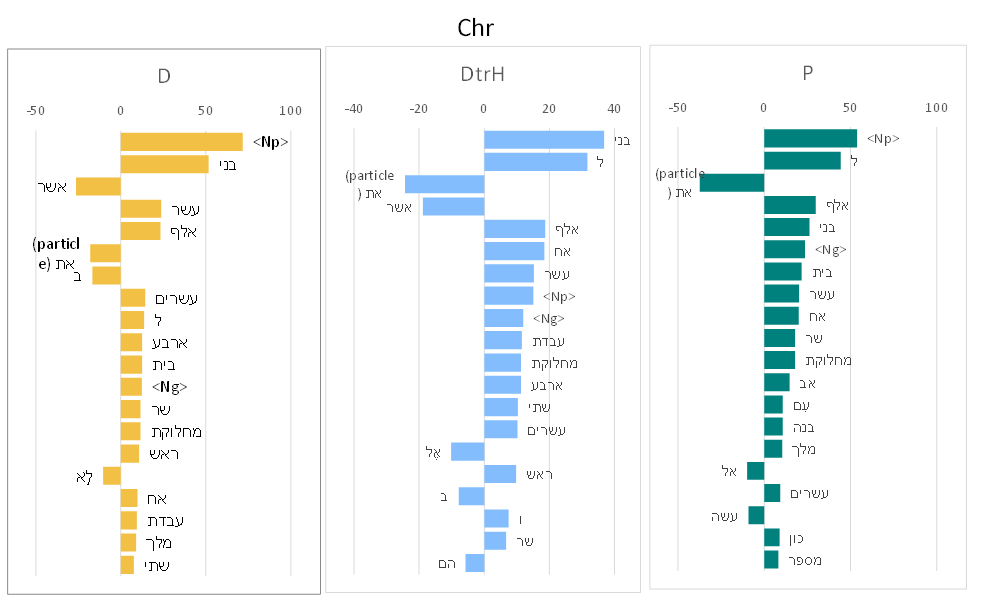  (E) | 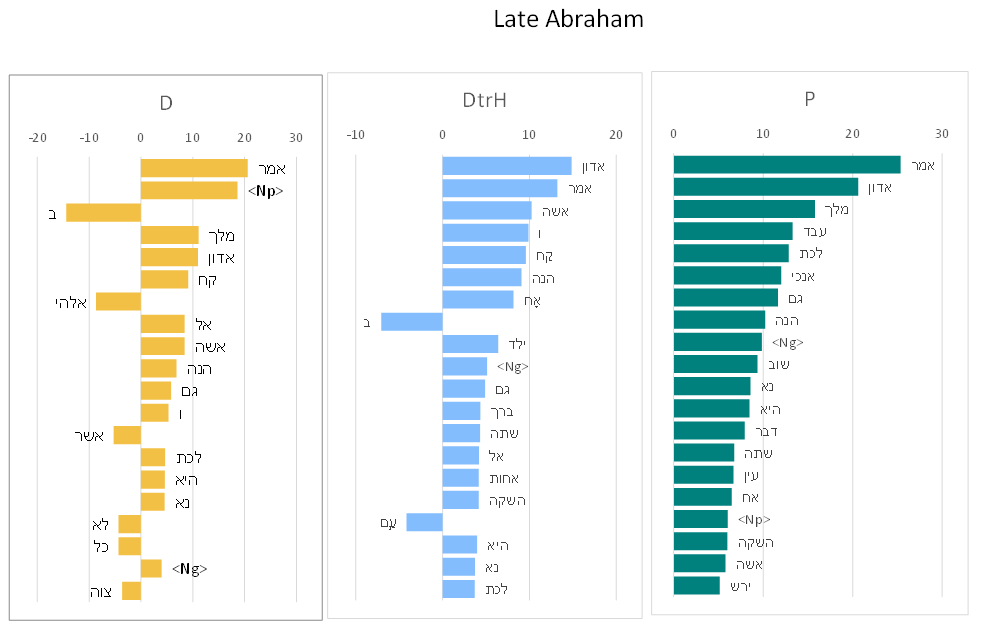  (F) |
| 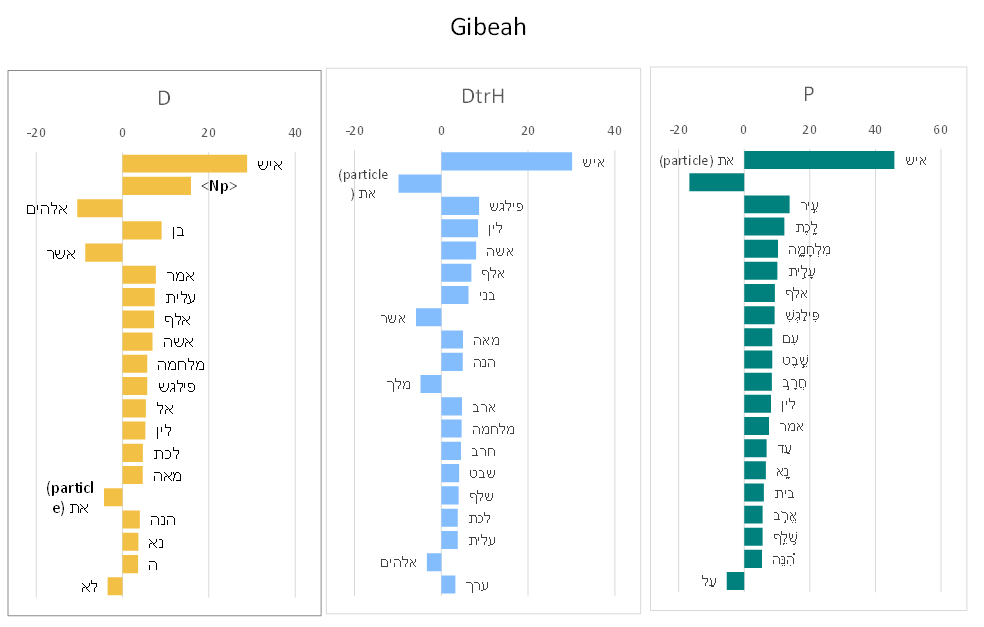  (G) | 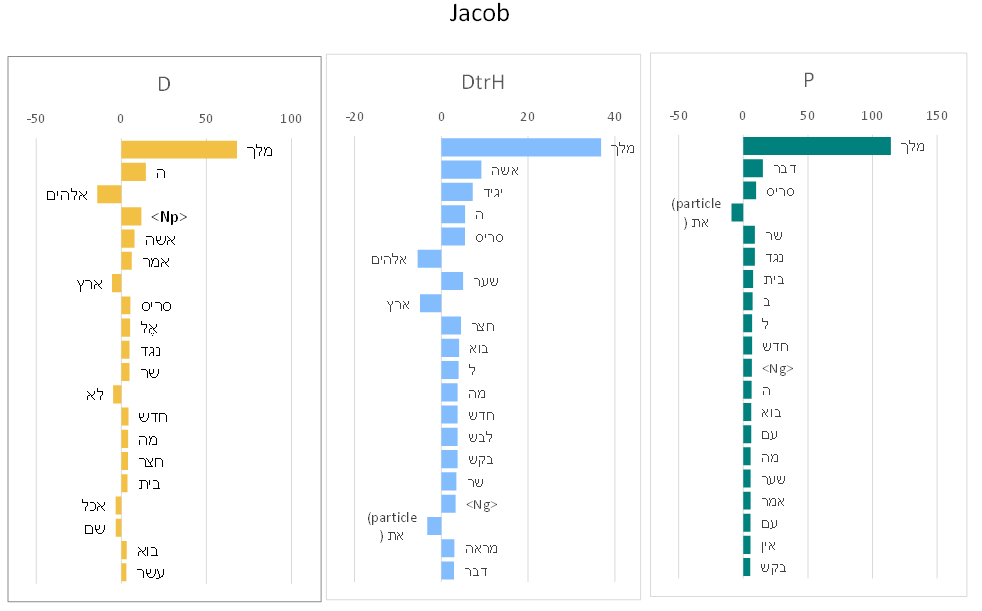  (H) |
| 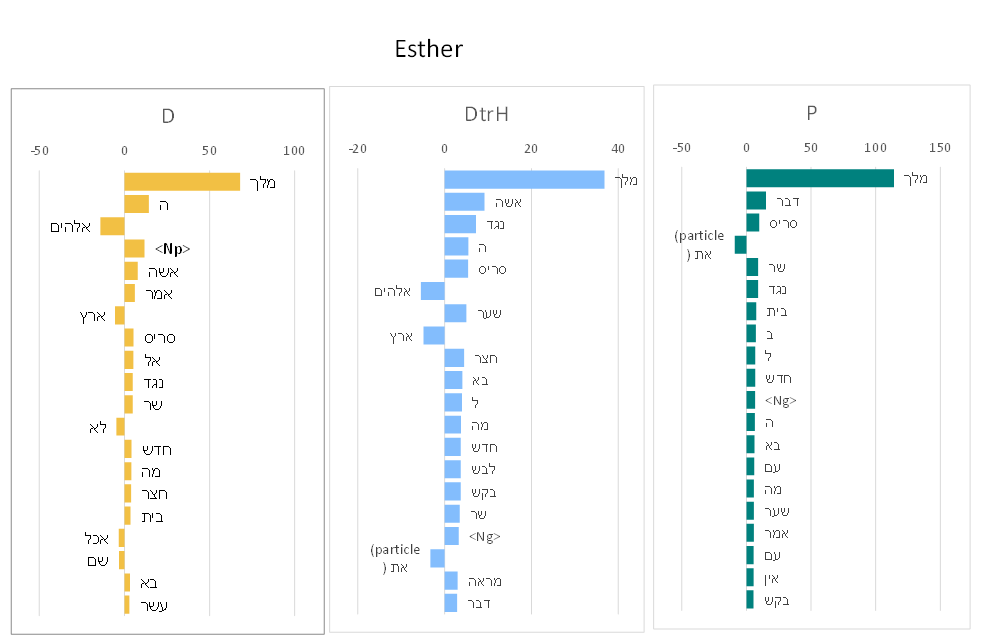 (I) | 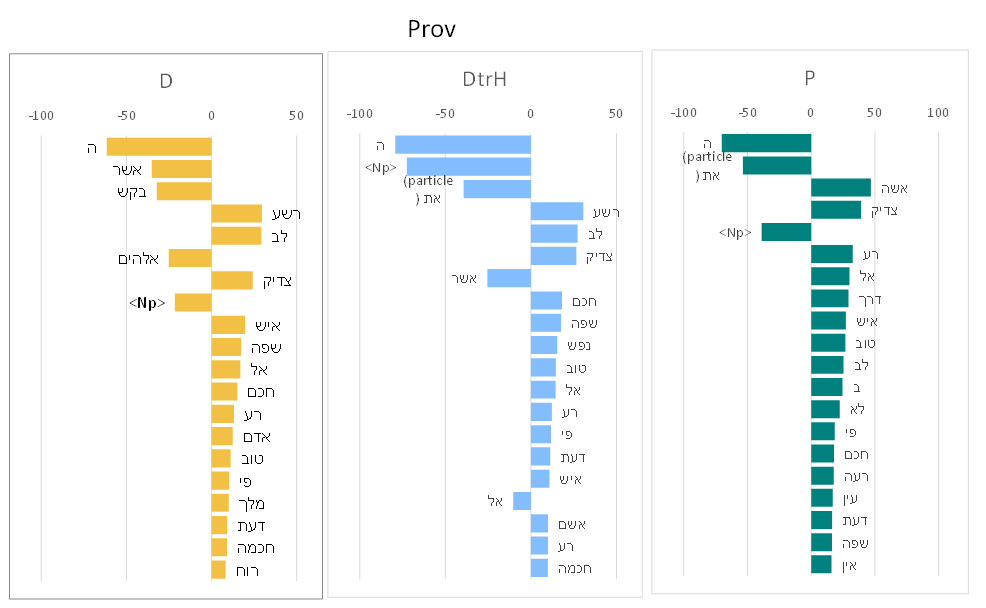 (J) |

**Figure S7.** Discriminating lemmas between the biblical texts and the D, DtrH, and P ground-truth corpora. Each graph lists the top 20 lemmas that are the most important of distinguishing the current text from these three corpora. The lemmas were selected by the HC algorithm and are ordered by their significance level (log p-value). The biblical texts compared were: (A) Deut4; (B) Lev26; (C) Ark 1 (D) Ark 2; (E) Chr; (F) Late Abraham; (G) Gibeah (H) Early Jacob; (I) Esther; (J) Prov.

# The Accuracy and Robustness Evaluation

In order to test the robustness of the HC score with respect to changes in the distribution of the lemmas, and its effect on the attribution process we performed a few tests to study different aspects of the method. The tests aimed at tracing a possible setting in the textual data which could influence the accuracy of the HC-attribution method and its robustness.

**Test 1: Robustness with respect word statistic**

First, the connection between the text length to the attribution process was examined. The ***HC-accuracy is closely related to the word statistic***, and thus the length of the text is expected to plays a significant role in accuracy evaluation. We tested the HC-attribution accuracy with respect to texts with 5, 10, 30, 50, 75, 100, 125, 150, 175, and 200 verses. In order to simulate texts of this length, small-length texts were artificially created by randomly sampling a pre-selected number of verses from the existing chapters. We evaluated the HC-attribution accuracy for these new texts, and the p-value for the attribution was calculated, and compared against the ground-truth labeling. This process was executed for bootstrap 1500 iterations. Last, the mean accuracy was calculated. In Figure S8 the accuracy is plotted with respect to the length of the text. One can see that while for 5 verses, the accuracy is not sufficiently high (63%), for 30 verses, the accuracy is already reasonable (83%), and for texts above 175 words, the accuracy is above 90%, see Figure S8.

**Figure S8.** **Testing the accuracy of texts with different lengths**. The x-axis corresponds to the length of the document measured in number of S. The blue dots indicate the mean value across the bootstrap iterations, while the intervals indicate the corresponding standard deviation.

In order to test the robustness of the method with respect to the theme of the chapters, we extracted all the existing unique words in the 50 chapters (after the lemmatization process). Let denote the number of the unique words by $N$. We simulated different word statistics by performing 100 bootstrap simulations, where each time we randomly selected (with replacement) a set of $N$ words. Thus, artificial texts were created, from the original 50 chapters, by using only the $N$ random words (if a word existed more than once, then a number was added to its name, and the word frequency statistics remained the same as the original word). We applied the HC-attribution method on the new artificial 50 texts. We quantified the attribution accuracy in the following manner. Using the success rate of the attribution in the 100 bootstrap iterations, we calculate the p-value of attribution of a given text to each of the three corpora. In total, the standard deviation of the attribution accuracy with respect to changing word statistics is 4%. This fact indicates the attribution process is robust and does not depend on certain word distribution (or on a group of words, that can be theme-dependent).

**Test 2: Robustness with respect to reference data**

Next, we tested the extent to which the ***text attribution is reference data-dependent***. Specifically, if the reference corpora would vary, would the attribution change? In order to check this, we evaluate the accuracy via k-fold cross-validation, with $k=4$. We randomly divided the reference data into four groups of 13 chapters each. This was done by dividing each corpus into four groups separately with a total number of chapters being $\left\lfloor\left. \frac{9}{4} \right\rceil\right.+\left\lfloor\left. \frac{19}{4} \right\rceil\right.+\left\lfloor\left. \frac{22}{4} \right\rceil\right.=13$ . In each iteration, we randomly selected one group to serve as the validation data, while the remaining three groups were used as the reference ground-truth data (i.e., 39 chapters). Next, we attributed each document from the validation data to one of the existing corpora (D, DtrH, or P) with respect to the 39 chapters. Executing 130 Monte Carlo simulations resulted in an accuracy mean of 85.8% with a standard deviation of 5% across repetitions.

**Test 3: Robustness with respect to n-grams**

In addition, we verified that the ***accuracy is robust with respect to the selected feature***, i.e., single term, bigram, trigram.

We will denote as n-grams as a combination of several consecutive lemmas. For example, ,וישמע אשר צוה, אתה ו, are bi-grams. How do we construct the bi-grams and trigrams? Once the lemmas are extracted by the OSHB, a unique number is associated with each lemma, referring to its lemma form. Thus, some words can be fragmented in to the main word, prefix and/or suffix, each result in several unique number id. For example, והכהנים produces three consecutive lemmas and results in thee id: c/d/3548. Certain lemmas like proper nouns are later replaces by a code, say <PN>. When evaluating lemma 2 and 3 grams, we use all lemmas in our list post replacement. In this work we denote as n-grams a combination of several consecutive lemmas. For example, ,ויאמר ידבר אלהים, מארץ, are bi-grams, while והכהנים, אשר הוצאתיך מ, לא תעשה פסל are trigram.

We repeated the HC-attribution process with either bigram, or trigram base feature of the HC (by joining consecutive lemmas). We summarized the results in Table S10. We see that the accuracy in all the cases is relatively good. We also notice that using bigrams slightly improves the attribution accuracy, and also results with no FN. However, in our main experiment, we preferred using a single term as the feature, due to its simplicity and explicability. Testing various n-grams was in fact a pillar stone in the attribution process that proves that the attribution is not word-dependent and not even theme-dependent. A key feature of the HC method is a set of separating features. We show that one can characterize the author not only by a set of words but also by using bigrams or trigrams.

**Table S10.** **Attribution accuracy of the ground truth vs. n-gram features used**

| Used Feature | Attribution Accuracy of the Ground Truth | FN Rate |
| --- | --- | --- |
| Bigrams | 88% | 0 |
| Trigrams | 74% | 4% (2 out of 50) |
| Single term, bigram, and trigram | 84% | 0 |

**SI References**

1. Schniedewind, W. M., How the Bible Became a Book: The Textualization of Ancient Israel (Cambridge: Cambridge University Press; 2004.
2. Kratz, R. G., The composition of the Narrative Books of the Old Testament (A&C Black); 2005.
3. Schmid, K., The Old Testament: A Literary History (Fortress Press); 2012.
4. Romer, T., The so-called Deuteronomistic history: a sociological, historical and literary introduction (London); 2005.
5. Finkelstein, I., Essays on biblical historiography: from Jeroboam II to John Hyrcanus I. Essays on Biblical historiography. 2022; 1–601.
6. Stamatatos, E. A survey of modern authorship attribution methods. J. Am. Soc. for information Sci. Technol.; 2009; 60 (32):538-556.
7. Swain, S., Mishra, G., & Sindhu, C., Recent approaches on authorship attribution techniques—An overview. In 2017 Int. Conf. of Electronics, Communication and Aerospace Technology, 2017; 1**:**557-566.‏
8. Tyo, J., Dhingra, B., & Lipton, Z. C., Valla: Standardizing and Benchmarking Authorship Attribution and Verification Through Empirical Evaluation and Comparative Analysis. In Proceedings of the 13th International Joint Conference on Natural Language Processing and the 3rd Conference of the Asia-Pacific Chapter of the Association for Computational Linguistics, 2023; 649-660.‏
9. Portnoy, S. L., Petersen, D. L., Biblical texts and statistical analysis: Zechariah and beyond. J. Biblical Lit. 1984; 103:11–21.
10. Holmes, D. I., Authorship attribution. Comput. Humanit. 1994; 28:87–106.
11. Stamatatos, E., A survey of modern authorship attribution methods. J. Am. Soc. for information Sci. Technol. 2009; 60:538–556.
12. Jockers, M. L., Witten, D. M., A comparative study of machine learning methods for authorship attribution. Lit. Linguist. Comput. 2010; 25:215–223.
13. Neal, T., Sundararajan, K., Fatima, A., Yan, Y., Xiang, Y., & Woodard, D, Surveying stylometry techniques and applications. ACM Computing Surveys (CSuR), 2017; 50(6):1-36.
14. Koppel, M. Akiva, N., Dershowitz, I., Dershowitz, N., Unsupervised decomposition of a document into authorial components in Proc. of the 49th Annual Meeting of the Association for Computational Linguistics: Human Language Technologies. 2011; 1356–1364.
15. Dershowitz, I., Akiva, N., Koppel, M., Dershowitz, N., Computerized source criticism of biblical texts. J. Biblical Lit. 2015; 134:253–271.
16. Shrestha, P., Sierra, S., González, F. A., Montes, M., Rosso, P., & Solorio, T. Convolutional neural networks for authorship attribution of short texts in Proceedings of the 15th Conference of the European Chapter of the Association for Computational Linguistics, 2017: 2: 669–674.
17. Wang, L., News authorship identification with deep learning in Conference and Labs of the Evaluation Forum, Portugal 2016.
18. Kipnis, A., Higher criticism for discriminating word-frequency tables and authorship attribution. The Annals Appl. Stat. 2022; 16:1236–1252.
19. Donoho, D. L., Kipnis, A., Higher criticism to compare two large frequency tables, with sensitivity to possible rare and weak differences. The Annals Stat. 2022; 50:1447–1472.
20. Otto, E., Deuteronomium 1 - 11. Teilbd. 1: 1,1 - 4,43 (Freiburg Basel Wien Herder 2012) 2012: deut, 2.
21. Preuss, H.D., Deuteronomium. (Wissenschaftl. Buchgesellschaft) 1982; 164.
22. Dietrich, W., Mathys, H. P., Romer, T., Smend, R., Die Entstehung des Alten Testaments. (Kohlhammer Verlag) 2014.
23. Stamatatos, E., et al., Overview of the authorship verification task at PAN 2022. In CEUR workshop proceedings 2022; **3180**:2301-2313.
24. Brown, I. V., The higher criticism comes to America, 1880—1900. J. Presbyt. Hist. Soc. (1943-220 1961) 1960; 193–212.
25. Nihan, C., From Priestly Torah to Pentateuch: A study in the composition of the Book of Leviticus. (Mohr Siebeck) 2007; 25.
26. Schmid, K, The Neo-Documentarian Manifesto: A Critical Reading. Journal of Biblical Literature, 2021; 140 (3):461-479.‏
27. Faigenbaum-Golovin, S., Shaus, A., Sober, B., Levin, D., Na’aman, N., Sass, B., et al. Algorithmic handwriting analysis of Judah’s military correspondence sheds light on composition of biblical texts. Proceedings of the National Academy of Sciences, 2016; **113** (17):4664-4669.
28. Faigenbaum-Golovin, S., Shaus, A., Sober, B., Turkel, E., Piasetzky, E., Finkelstein, I. Algorithmic handwriting analysis of the Samaria inscriptions illuminates bureaucratic apparatus in biblical Israel. Plos One 2020; 15 (1).‏
29. Li, J. S., Chen, L. C., Monaco, J. V., Singh, P., & Tappert, C. C., A comparison of classifiers and features for authorship authentication of social networking messages. Concurrency and Computation: Practice and Experience, 2017; 29(14): 3918.‏
30. Oliveira Jr, W., Justino, E., Oliveira, L. S. Comparing compression models for authorship attribution. Forensic science international, 2013; 228 (1-3):100-104.‏
31. Murauer, B., & Specht, G., Developing a benchmark for reducing data bias in authorship attribution. In Proc. of the 2nd Workshop on Eval. and Comparison of NLP Sys. 2021; 179-188.‏
32. Mosteller, F., Wallace, D. L., Inference in an authorship problem: A comparative study of discrimination methods applied to the authorship of the disputed federalist papers. J. Am. Stat. Assoc. 1963; 58:275–309.
33. Open scriptures Hebrew Bible project (2019). Original work of the Open Scriptures Hebrew Bible available at https://github.com/openscriptures/morphhb (Accessed May 8, 2024).
34. Kipnis, A., Donoho, D. L., Two-sample testing of discrete distributions under rare/weak perturbations in IEEE Int. Sym. on Inf. Theory (ISIT) 2021; 3314–3319.
35. Donoho, D. L., Jin, J., Higher criticism for detecting sparse heterogeneous mixtures. The Annals Stat. 2004; 32:962–994.
36. Donoho, D. L., Jin, J., Higher criticism thresholding: optimal feature selection when useful features are rare and weak. Proc. Natl. Acad. Sci. 2008; 105:14790–14795.
37. Bevendorff, J., et al., Overview of PAN 2020: Authorship verification, celebrity profiling, profiling fake news spreaders on Twitter, and style change detection. In Experimental IR Meets Multilinguality, Multimodality, and Interaction: 11th International Conference of the CLEF Association, Proc. 2020; 11**:**372-383.‏
38. Finkelstein, I., Romer, T., The historical and archaeological background behind the old Israel ark narrative. Peeters, 2020.
39. Japhet, S., I and II Chronicles: a commentary. (Westminster John Knox Press) 1993.
40. Abadie, P., Introduction à l’Ancien Testament. (Labor et fides) 2009; 49.
41. Finkelstein, I., Romer, T., Comments on the historical background of the Abraham narrative. between “Realia” and “Exegetica”. Hebr. Bible Anc. Isr. 2014; 3:3–23.
42. Becker, U., Richterzeit und Königtum: Redaktionsgeschichtliche Studien zum Richterbuch. (Walter de Gruyter) 1990; 192.
43. Lanoir, C., “Juges”, in P. Abadie, Introduction à l’Ancien Testament. (Labor et fides) 2009; 49.
44. Knauf, E. A., “L “historiographie Deutéronomiste” existe-t-elle”, in Israël Construit Son Histoire. L’Historiographie Deutéronomiste à La Lumière Des Recherches Récentes, ed. by Albert de Pury, Thomas Römer, and Jean-Daniel Macchi (Genève: Labor et Fides) 1996: 409–418
45. Finkelstein, I., Romer, T., Comments on the historical background of the Jacob narrative in Genesis. Zeitschrift für die alttestamentliche Wissenschaft 2014; 126:317–338.
46. Macchi, J. D., Le Livre d’Esther, Commentaire de l’Ancien Testament XIV (Genève: Labor et Fides) (2016).
47. Steymans, H. U., Verheißung und Drohung: Lev 26, in: H.-J. Fabry et H.-W. Jüngling (éd), Levitikus als Buch (BBB 119), Berlin - Bodenheim b. Mainz: Philo, 1999; 263-307.
48. Stackert, J., Rewriting the Torah: literary revision in Deuteronomy and the Holiness Legislation 52 (Forschungen zum Alten Testament,), Tübingen: Mohr Siebeck, 2007.
49. Nihan, C., Heiligkeitsgesetz und Pentateuch. Traditions- und kompositionsgeschichtliche Aspekte von Levitikus 26, in: F. Hartenstein et K. Schmid (éd), Abschied von der Priesterschrift? Zum Stand der Pentateuchdebatte (Veröffentlichungen der Wissenschaftlichen Gesellschaft für Theologie 40), Leipzig: Evangelische Verlagsanstalt 2015; 186-218.
50. Rost, L., Die Überlieferung von der Thronnachfolge Davids. (Kohlhammer) 2011; 42.
51. Campbell, A.F., I Samuel. (Wm. B. Eerdmans Publishing), 2003.
52. Bodner, K., Ark-Eology: Shifting Emphases in ‘Ark Narrative’Scholarship. Curr. biblical research, 2006; 4:169–197.
53. Signoriello, D. J., Jain, S., Berryman, M. J., Abbott, D., Advanced text authorship detection methods their application to biblical texts”in Proc. SPIE. 2005; 6039: 163–175
54. Basili, R., Nissim, M., Satta, G., Proc. of the Fourth Italian Conf. on Comp. Linguistics CLiC-it 2017 (Accademia University Press) 2016.
55. Schwartz, R., Tsur, O., Rappoport, A., Koppel, M., Authorship attribution of micro-messages in Proc. of the 2013 Conf. on empirical methods in natural language proc. 2013; 1880–1891.
56. Kapočiūtė-Dzikienė, J., Utka, A., Šarkutė, L., Authorship attribution and author profiling of Lithuanian literary texts in The 5th Workshop on Balto-Slavic Natural Language Proc. 2015; 96–105.
57. Ellegård, A., A Statistical method for determining authorship: the Junius Letters, 1962; 13:1769-1772.
58. Brinegar, C. S., Mark Twain and the Quintus Curtius Snodgrass letters: A statistical test of authorship. J. Am. Stat. Assoc. 1963: 58:85–96.
59. Thisted, R., Efron, B., Did Shakespeare write a newly-discovered poem? Biometrika 1987; 74:445–455.
60. Mikros, G.K. and Argiri, E.K. Investigating topic influence in authorship attribution, in Proc. of the SIGIR 2007 Int. Workshop on Plagiarism Analysis, Authorship Identification, and Near-Duplicate Detection 2007.
61. Porter, M. F., An algorithm for suffix stripping. Program 1980.
62. Lovins, J. B., Development of a stemming algorithm. Mech. Transl. Comput. Linguist. 1968; 11:22–31.
63. Jivani, A. G., et al., A comparative study of stemming algorithms. Int. J. Comp. Tech. Appl 2011; 2:1930–1938.
64. Tsarfaty, R., Seker, A., Sadde, S., Klein, S., What’s wrong with Hebrew NLP? and how to make it right. arXiv 2019. arXiv:1908.05453. (Accessed May 8, 2024).
65. Qi, P., Dozat, T., Zhang, Y., Manning, C. D., Universal dependency parsing from scratch. arXiv 2019. arXiv:1901.10457 (Accessed May 8, 2024).
66. Analytical tools for Hebrew texts. Dicta. Available at https://dicta.org.il/ (Accessed May 8, 2024).
67. Stamatatos, E., On the robustness of authorship attribution based on character n-gram features, Journal of Law and Policy, 2013: 21(2):421–439.
68. Potha, N., Stamatatos, E. “An Improved Impostors Method for Authorship Verification”, in International Conference of the Cross-Language Evaluation Forum for European Languages, 2017:138–144.
69. Salton, G., Automatic information organization and retrieval, McGraw-Hill, New York. 1968
70. Spärck Jones, K., A statistical interpretation of term specificity and its application in retrieval, Journal of Documentation, 1972:28:11–21.
